# Biomimetic Layered Double Hydroxide-Molybdenum Disulfide Encapsulated with Bovine Serum Albumin: A Multifaceted Nanotherapy for Inflammatory Bowel Disease

**DOI:** 10.34133/bmr.0369

**Published:** 2026-05-18

**Authors:** Yijie Xie, Qiwen Jiang, Huabing Huang, Xinyuan Zhang, Xiaoyi Zheng, Yunyi Yang, Shige Wang, Jiulong Zhao, Zhaoshen Li

**Affiliations:** ^1^ Department of Gastroenterology, Shanghai Institute of Pancreatic Diseases, Changhai Hospital; National Key Laboratory of Immunity and Inflammation, Naval Medical University, Shanghai 200433, P. R. China.; ^2^ School of Materials and Chemistry, University of Shanghai for Science and Technology, Shanghai 200093, P. R. China.

## Abstract

Inflammatory bowel disease (IBD) remains a therapeutic challenge due to its ongoing inflammation and the limited availability of effective local therapies. This study developed a biomimetic nanocomposite, layered double hydroxide-molybdenum disulfide (LDH-MoS_2_ [LM]) nanosheets encapsulated with bovine serum albumin (abbreviated as LM@BSA), with the aim of enhancing reactive oxygen species scavenging efficiency and improving colloidal stability while favoring lesion-associated localization and local retention in inflamed colonic tissue. In a dextran sulfate sodium-induced colitis mouse model, oral administration of LM@BSA nanocomposite significantly improved disease outcomes, as shown by reduced colon shortening, less body weight loss, and decreased levels of pro-inflammatory cytokines. Mechanistic analysis demonstrated that the LM@BSA nanocomposite inhibits ferroptosis by protecting mitochondrial structure and increasing glutathione peroxidase 4 expression. Transcriptomic data showed that the LM@BSA nanocomposite helps restore intestinal balance by down-regulating the phosphoinositide 3-kinase–protein kinase B signaling pathway, regulating extracellular matrix remodeling, and supporting immune pathway stability. Moreover, the LM@BSA nanocomposite corrected gut microbiota dysbiosis by increasing the abundance of beneficial *Bacteroidales* and reducing pro-inflammatory *Desulfovibrionales*. These findings suggest that the LM@BSA nanocomposite offers a promising and comprehensive multifaceted nanotherapeutic approach for IBD by modulating oxidative stress, ferroptosis, immune dysregulation, and gut microbiota imbalance.

## Introduction

Inflammatory bowel disease (IBD), primarily including Crohn’s disease and ulcerative colitis, is characterized by chronic inflammation of the gastrointestinal tract [1]. The global incidence and prevalence of IBD are rising steadily, representing a substantial and growing burden on healthcare systems worldwide [2]. The pathogenesis of IBD is complex and is driven by interactions among genetic predisposition, environmental influences, imbalance of gut microbiota, and abnormal immune responses [3,4]. This leads to a compromised intestinal barrier, persistent inflammation, and subsequent tissue injury [5]. Oxidative stress is a key driver of disease progression, where excessive reactive oxygen species (ROS) damage cellular components and exacerbate mucosal injury [6,7]. Furthermore, ferroptosis, a novel form of iron-dependent regulated cell death, has been implicated in the loss of epithelial cells in IBD, linking oxidative stress and metabolic dysregulation to tissue damage [8,9].

Current therapeutic strategies for IBD, while having improved patient outcomes, face considerable limitations. Conventional agents like aminosalicylates and corticosteroids are effective for symptom control in mild-to-moderate disease yet often fail to induce long-term remission and are associated with side effects such as osteoporosis [10,11]. Immunosuppressants like azathioprine are used for maintenance but increase infection risks [12]. Biologics targeting tumor necrosis factor or integrins can induce mucosal healing [13,14]. However, primary nonresponse and secondary loss of response due to immunogenicity remain major challenges [15,16]. While small-molecule Janus kinase inhibitors offer oral administration and rapid efficacy, concerns regarding thromboembolic events and other potential side effects persist [17]. Having emerged recently, fecal microbiota transplantation aims to restore gut microbial homeostasis and shows promise, particularly in ulcerative colitis [18,19], though standardized protocols and long-term efficacy data are lacking [20]. Nevertheless, existing therapies primarily focus on systemic immunosuppression and inadequately address localized pathological factors like oxidative stress and ferroptosis, leaving room for disease relapse and incomplete mucosal healing.

Advancements in nanotechnology have opened new avenues for IBD treatment by enabling targeted interventions. Nanomaterials facilitate precise drug delivery to inflamed intestinal sites, minimizing systemic exposure and side effects [21,22]. Nanozymes, which mimic natural enzyme activities, can act on many targets, such as effectively scavenge ROS and alleviate oxidative stress [23,24]. Molybdenum-based nanostructures, such as nanodots, have demonstrated potent antioxidant and anti-inflammatory effects in models of gut inflammation, with protective roles also established in other disease contexts like kidney injury, underscoring their therapeutic potential [25,26]. Layered double hydroxides (LDHs) serve as excellent oral delivery vehicles due to their high drug-loading capacity and pH-responsive release profiles in the gastrointestinal tract [27,28]. Notably, LDH-based nanozymes have emerged as a versatile platform with inherent enzyme-like activities for biomedical applications [29]. It has been reported that composites incorporating molybdenum disulfide (MoS_2_) show considerable catalytic activities against ROS [30,31]. Inspired by the superior catalytic performance achieved through multimetallic synergy in advanced nanozyme designs [32,33], these multifunctional nanohybrids can simultaneously target inflammatory pathways and cell death mechanisms, including ferroptosis. Moreover, surface modifications with biomolecules like proteins can improve nanoparticle stability and enhance their interaction with inflamed mucosa, thereby favoring local retention [34]. Such sophisticated nanosystems also positively modulate the gut microbiota, promoting beneficial species and suppressing pathobionts [35,36].

Building on these advances, we developed a novel oral nanozyme, LDH-molybdenum disulfide nanosheets encapsulated with bovine serum albumin (BSA) (LDH-MoS_2_@BSA, abbreviated as LM@BSA), tailored as a multifaceted nanotherapeutic agent for IBD. This system is engineered by integrating LDH with MoS_2_ (LDH-MoS_2_ [LM]), followed by a BSA coating to enhance its colloidal stability and biocompatibility [37,38]. A key action mechanism is its potent ROS-scavenging capability, primarily conferred by the Mo component, which effectively mitigates oxidative stress and associated lipid peroxidation, and helps to protect intestinal epithelial cells from ferroptosis. In IBD mice models, treatment with the LM@BSA nanocomposite has demonstrated marked therapeutic benefits, including a marked reduction in pro-inflammatory cytokine levels and promotion of epithelial barrier restoration. Furthermore, the nanozyme contributes to a healthier gut microbiota composition. Comprehensive biosafety evaluations have indicated low systemic toxicity and excellent tissue compatibility, supporting its potential for further clinical development. By concurrently addressing oxidative stress, programmed cell death, and inflammation, the LM@BSA nanocomposite represents a multifaceted therapeutic strategy that operates without broad immunosuppression, showing promise for improving long-term remission rates in IBD.

## Materials and Methods

### Materials

Sodium hydroxide (NaOH), ammonium tetrathiomolybdate [(NH_4_)_2_MoS_4_], magnesium nitrate hexahydrate [Mg(NO_3_)_2_·6H_2_O], and aluminum nitrate nonahydrate [Al(NO_3_)_3_·9H_2_O] were obtained from Aladdin Bio-Chem Technology Co., Ltd. (Shanghai, China). BSA was purchased from Yeasen Biotechnology (Shanghai, China). Total glutathione peroxidase assay kit and the ROS-sensitive probe 2′,7′-dichlorodihydrofluorescein diacetate (DCFH-DA) probe were obtained from Beyotime Biotechnology (Shanghai, China). L929 was provided by the Institute of Biochemistry and Cell Biology, Chinese Academy of Sciences (Shanghai, China). Dulbecco’s modified Eagle medium (DMEM) and phosphate-buffered saline (PBS), simulated gastric fluid (SGF), and simulated intestinal fluid (SIF) were purchased from Corning Life Sciences (Shanghai, China). Cell Counting Kit-8 (CCK-8) was supplied by Beyotime Biotechnology (Shanghai, China). Male C57BL/6 mice (4 to 6 weeks, 20 to 25 g) were acquired from GemPharmatech Co., Ltd. (Nanjing, Jiangsu, China) and maintained in accordance with the guidelines of the Ministry of Health of the People’s Republic of China. Enzyme-linked immunosorbent assay (ELISA) kits for tumor necrosis factor-alpha (TNF-α), interleukin-6 (IL-6), and interleukin-1 beta (IL-1β) were purchased from Elabscience Biotechnology (Wuhan, China). Calcein-AM/PI Live/Dead and Annexin V-FITC/PI apoptosis detection kits were obtained from Yisheng Biotechnology (Shanghai, China) and Vazyme Biotechnology (Nanjing, China), respectively. Caerulein was procured from MedChemExpress (Shanghai, China).

### Preparation and characterization

LM@BSA nanosheets were fabricated via a hydrothermal synthesis approach, following the detailed procedures below: First, 2 stock solutions were pre-prepared: a 0.15 mol/l NaOH solution and a 5 mg/ml (NH_4_)_2_MoS_4_ solution. Subsequently, 0.164 g of Mg(NO_3_)_2_·6H_2_O and 0.060 g of Al(NO_3_)_3_·9H_2_O were accurately weighed and dissolved in 10 ml of deionized (DI) water to form a homogeneous mixture. The pre-prepared 0.15 mol/l NaOH solution (20 ml) was rapidly added to the above mixture, followed by continuous stirring for 1 h. The resulting mixture was centrifuged at 8,000 rpm for 5 min, and the supernatant was discarded to collect the bottom precipitate.

Next, 25 ml of DI water and 10 ml of the 5 mg/ml (NH_4_)_2_MoS_4_ solution were added to the container with the precipitate, and the mixture was stirred until the precipitate was completely dissolved. The dissolved solution was transferred to a magnetic stirrer for continuous stirring over 90 min and then transferred into a 100-ml stainless steel autoclave (lined with polyphenylene). The autoclave was placed in an oven and maintained at 180 °C for 12 h to conduct the hydrothermal reaction. After the reaction, the autoclave was allowed to cool down to room temperature naturally. The internal solution was taken out, centrifuged at 8,000 rpm for 10 min, and the collected precipitate was washed 3 times with DI water. The washed precipitate was freeze-dried in a freeze dryer for 12 h to obtain a black powdery product, denoted as LM, which was then finely ground for subsequent use.

For BSA modification, 10 mg of the ground LM powder was dispersed in 10 ml of DI water via ultrasonic treatment using an ultrasonic cell disruptor (power: 640 W; total duration: 40 min; working cycle: 3 s on/5 s off). Then, 250 mg of BSA was added to the ultrasonicated dispersion, and the mixture was shaken vigorously to ensure uniform mixing. Further ultrasonic modification was performed under the following parameters: power of 480 W, duration of 30 min per cycle, and four cycles in total (working cycle: 3 s on/5 s off). The modified dispersion was centrifuged at 13,000 rpm for 10 min, and the supernatant was discarded. The collected precipitate was washed twice with DI water, and 2 ml of DI water was added to the final precipitate. After another freeze-drying process, the obtained freeze-dried powder was the target LM@BSA nanocomposite.

Morphological characterization was performed using transmission electron microscopy (TEM, JEOL JEM-2100F) and scanning electron microscopy (SEM, Zeiss Sigma 300). Samples were prepared by depositing ethanol-diluted MoS_2_ or LM@BSA nanocomposite suspensions onto copper grids (TEM) and mica plates (SEM), respectively. Hydrodynamic diameters and colloidal stability of the LM@BSA nanocomposite were assessed via dynamic light scattering (DLS, Malvern Nano ZS90) in DI water, saline, PBS, and DMEM at specified time intervals, with the Tyndall effect confirming dispersion stability. Surface charge was determined by zeta potential measurements in aqueous suspension. Chemical composition and crystallinity were evaluated using x-ray photoelectron spectroscopy (XPS, Thermo Scientific K-Alpha) and x-ray diffraction (XRD, Rigaku D/max-2200), respectively. Molecular structures were analyzed by Fourier transform infrared (FTIR) spectroscopy (Nicolet Nexus 470).

### In vitro biocompatibility and intracellular ROS scavenging studies

In vitro biocompatibility was evaluated via assessments of blood compatibility and cytotoxicity. Specifically, the impact of the LM@BSA nanocomposite on cell viability was first investigated using a cytotoxicity assay, following the protocol described below: L929 cells were seeded into 96-well plates at a density of 8,000 cells per well and incubated for 24 h in a humidified atmosphere containing 5% CO_2_ at 37 °C. Subsequently, LM@BSA nanocomposites were dissolved in DMEM to prepare a series of working solutions with concentrations of 0, 1, 5, 10, 25, 50, and 100 μg/ml (100 μl per well), which were then cocultured with L929 cells for an additional 24 h. After removing the old culture medium, each well was replenished with 90 μl of fresh DMEM supplemented with 10 μl of CCK-8 reagent, followed by incubation for 2 h. The absorbance of each well was measured at 450 nm using a microplate reader, and the cell viability was calculated accordingly. For visualization of cell viability, L929 cells were washed 3 times with PBS after the aforementioned treatments, then stained with a calcein-AM/PI Live/Dead staining kit. Stained cells were observed under a fluorescence microscope (Leica DM IL LED Inverted Laboratory Microscope, Leica, Germany).

Tumor cell line RAW 264.7 (RAW264.7) macrophages and the normal human colonic mucosa epithelial cell line (NCM-460 cells) were cultured in Roswell Park Memorial Institute 1640 Medium (RPMI-1640) (10% fetal bovine serum [FBS]) and DMEM (10% FBS), respectively, at 37 °C with 5% CO_2_ to evaluate the LM@BSA nanocomposite’s protective effect against ROS-induced damage. To establish the ROS-induced inflammation model, RAW264.7 cells were treated with 100 mM H_2_O_2_ for 24 h, then the experimental group was incubated with LM@BSA nanocomposite-containing medium for 6 h (the control group received medium alone); intracellular ROS levels were detected using 10 μM of DCFH-DA (30 min staining) via flow cytometry (488 nm excitation, 525 nm emission), fluorescence microscopy (with Hoechst 33342 nuclear staining), and confocal microscopy.

NCM-460 cells seeded in confocal dishes (5 × 10^4^ cells/dish) and cultured overnight were used to assess the LM@BSA nanocomposite’s ability to scavenge mitochondrial superoxide. A cellular inflammation model was induced by treating cells with 100 mM of H_2_O_2_ for 24 h, after which the experimental group was incubated with LM@BSA nanocomposite-containing medium for 6 h (the control group received medium alone). Mitochondrial superoxide was labeled with 5 μM of Mitochondrial Superoxide Indicator Red (MitoSOX Red, a superoxide-specific probe unaffected by other ROS/RNS, 510 nm excitation, 580 nm emission) for 10 min, and mitochondria were colabeled with 100 nM Mitochondrial Tracker Green (Mito-Tracker Green, 490 nm excitation, 516 nm emission) for 20 min at 37 **°**C. After PBS washing, fluorescence images were captured via confocal microscopy.

### In vitro intestinal barrier function study

To evaluate the intestinal barrier repair capacity of the LM@BSA nanocomposite, we utilized a mouse colon organoid model. Organoids were embedded in Matrigel and initially cultured in Wnt3a-supplemented normal colon organoid medium (NCOM) at 37 **°**C with 5% CO_2_. The experiment was divided into 3 groups with distinct treatments. To establish an in vitro injury model, organoids designated for the IBD and IBD + LM@BSA groups were stimulated with 50 ng·ml^−1^ TNF-α for 24 h. The control group was cultured in parallel in standard NCOM without any TNF-α stimulation. Following the 24-h stimulation period, the TNF-α-containing medium was removed from the IBD and IBD + LM@BSA groups. The IBD group was then cultured in fresh NCOM, while the IBD + LM@BSA group was cultured in NCOM containing the LM@BSA nanocomposite. All 3 groups were cultured for an additional 5 days to assess barrier repair. For analysis, the organoids were fixed in 4% paraformaldehyde–PBS. Immunofluorescence staining was performed to visualize tight junction proteins. This involved overnight incubation at 4 °C with primary antibodies against Zonula Occludens-1 (ZO-1), Claudin-1, and Occludin, followed by a 1-h incubation at room temperature with corresponding fluorescent secondary antibodies. Additionally, cell proliferation and apoptosis were assessed using keratin-like protein Ki-67 (Ki-67) and terminal deoxynucleotidyl transferase-mediated dUTP nick end labeling (TUNEL) staining, respectively, according to the manufacturer’s protocols. Nuclei were counterstained with 4′,6-diamidino-2-phenylindole (DAPI), and all samples were imaged using a fluorescence microscope to observe fluorescence intensity and structural changes.

### In vivo therapeutic evaluation study

Animal studies were approved by the Animal Ethics Committee of Ganzhou People’s Hospital (No. PJD2025-007-01). To investigate the in vivo therapeutic effects of the LM@BSA nanocomposite, a dextran sulfate sodium (DSS)-induced IBD mouse model was established using C57BL/6 mice. The mice were randomly assigned to 4 groups: a control group (*n* = 6, receiving regular drinking water), an IBD group (*n* = 6, receiving 3% w/v DSS in drinking water), an IBD + LM group (*n* = 6, receiving 3% w/v DSS in drinking water), and an LM@BSA + IBD group (*n* = 6, receiving 3% w/v DSS in drinking water). For therapeutic intervention, LM or LM@BSA nanocomposite was administered via oral gavage on day 3 and day 5. Daily body weight changes were recorded to monitor disease progression. On day 8, all mice were euthanized, and colonic tissues were harvested immediately. Colonic length was measured, and mid-colonic segments were fixed in 4% paraformaldehyde for subsequent pathological assessment.

Distal colonic tissues from IBD group mice (day 8 postinduction) and healthy controls were collected for biological transmission electron microscopy (bio-TEM) and histological analysis. For bio-TEM: the colonic mucosal layer was dissected, fixed in 2.5% glutaraldehyde, dehydrated via gradient ethanol, embedded in epoxy resin, and sectioned into ultrathin slices. The sections were examined under a TEM to assess intestinal ultrastructure and LM@BSA nanocomposite localization. For histology: formalin-fixed colonic tissues were paraffin-embedded, cut into 5-μm sections, and stained with hematoxylin and eosin (H&E) and Masson’s Trichrome (Masson) staining. H&E staining evaluated inflammatory cell infiltration, while Masson’s staining assessed collagen deposition and fibrosis. Stained sections were visualized under light microscopy, and images were captured for intergroup comparison.

### Anti-inflammatory effects study

To explore the potential therapeutic mechanism of the LM@BSA nanocomposite in IBD, ELISA was performed to quantify inflammation-related cytokines in colonic tissues, alongside assessments of key ferroptosis-associated enzyme activity. For cytokine detection: 100 mg of colonic tissue per sample was homogenized in precooled PBS supplemented with protease inhibitors. Homogenates were centrifuged at 12,000 rpm for 15 min at 4 °C to collect supernatants. ELISA kits were used to measure levels of pro-inflammatory cytokines (IL-1β, TNF-α, and IL-6) in supernatants, with procedures strictly following kit instructions, including antibody coating, sample incubation, washing, color development, and absorbance measurement at 450 nm. Each sample was assayed in triplicate.

For glutathione peroxidase 4 (GPX4) activity assay: Colonic tissue supernatants (prepared as above) were used to determine GPX4 activity using a commercial assay kit from Nanjing Jiancheng Bioengineering Institute. Activity was calculated by monitoring the oxidation rate of nicotinamide adenine dinucleotide phosphate hydrogen (via absorbance changes at 340 nm) to evaluate the impact of the LM@BSA nanocomposite on this key ferroptosis-related enzyme. Assays were performed in triplicate for each sample.

### Disease activity index scoring

Daily observations were conducted on IBD model mice to record weight change, hematochezia, and diarrhea. The disease activity index was calculated by summing scores from 3 parameters: weight loss, fecal consistency, and fecal bleeding. Scores were documented to reflect cumulative disease severity.

### Histopathological analysis

Colonic tissue segments (approximately 5 mm) were fixed in 4% paraformaldehyde for 24 h, dehydrated via graded ethanol, and paraffin-embedded. Serial 5-μm sections were H&E stained following standard protocols. Histological scoring, performed blinded, evaluated inflammatory infiltration, crypt integrity, and ulceration extent (score range: 0 to 12, higher scores = more severe damage).

### Immunohistochemistry

Formalin-fixed paraffin-embedded (FFPE) colonic sections were deparaffinized in xylene (2 × 10 min) and rehydrated in graded ethanol (100%, 95%, 85%, and 75%; 5 min each). Antigen retrieval was done by boiling in citrate buffer (pH 6.0) for 15 min (microwave, 700 W). After cooling, sections were washed with PBS (pH 7.4; 3×5 min). Endogenous peroxidase was blocked with 3% H_2_O_2_ in methanol for 10 min (room temperature), followed by 3% BSA–PBS blocking for 30 min. Sections were incubated overnight at 4 °C with primary antibodies: anti-MPO (1:1,000, Abcam) and anti-TNF-α (1:800, Cell Signaling Technology). After PBS washes, horseradish peroxidase-conjugated goat anti-mouse immunoglobulin G (IgG; 1:2,000, Thermo Fisher) was applied for 50 min (room temperature). Color was developed with 3,3′-diaminobenzidine (Sigma-Aldrich) for 5 min, followed by hematoxylin counterstaining.

### Immunofluorescence

FFPE colonic sections underwent antigen retrieval (citrate buffer, pH 6.0; microwave boiling, 15 min) and blocking with 3% BSA in phosphate-buffered saline with Tween-20 (PBST, 0.1% Tween-20 in PBS) for 30 min (room temperature). Sections were incubated overnight at 4 °C with primary antibodies: anti-ZO-1 (1:1,000, Invitrogen) and anti-occludin (1:1,000, BD Biosciences) in 1% BSA–PBST. After PBST washes (3 × 5 min), CoraLite488-conjugated goat anti-rabbit IgG (1:500, Proteintech) was applied for 50 min (room temperature, dark). Nuclei were stained with DAPI (1 μg/ml, Sigma-Aldrich) for 10 min, and sections were mounted with anti-fade medium (Vectashield). Images were captured via confocal microscopy (Leica TCS SP8).

### 16S gene sequencing and analysis

Fecal samples were collected from individual mice using autoclaved sterile tubes on day 8 (immediately prior to euthanasia) and stored at −80 °C. The samples were shipped on dry ice to Shanghai Applied Protein Technology Co., Ltd. for 16S rRNA gene sequencing. Briefly, total genomic DNA was extracted from fecal samples using the CTAB/SDS method. Sequencing libraries were constructed following standard protocols and sequenced on an Illumina HiSeq 6000 platform in paired-end mode. Raw sequencing data were processed and analyzed on APT-BioCloud, including quality filtering, operational taxonomic unit clustering, and taxonomic annotation against the Silva reference database.

### RNA sequencing and transcriptomic analysis

Total RNA was extracted from colonic tissues using TRIZOL reagent, and samples with high integrity were selected for library construction. Sequencing libraries were prepared following standard Illumina protocols, including mRNA enrichment, cDNA synthesis, and adapter ligation. The resulting libraries were quantified and sequenced on an Illumina NovaSeq platform to generate paired-end reads. For bioinformatic analysis, raw data underwent quality control and adapter trimming, followed by alignment to the mouse reference genome. Gene expression levels were quantified, and differential expression analysis was performed to identify significantly altered genes. Functional enrichment analysis of these genes was carried out using GO and KEGG databases to elucidate the biological processes and pathways involved.

### In vivo biocompatibility and biosafety assessment study

To evaluate the in vivo biocompatibility and biosafety of the LM@BSA nanocomposite, mice were subjected to retro-orbital blood collection at the experimental endpoint. Serum was isolated by centrifugation at 3,000 rpm for 10 min and analyzed using an automated biochemistry analyzer with commercially available kits to measure hepatic and renal function indices, including creatinine (CREA), urea (UREA), aspartate aminotransferase (AST), alanine aminotransferase (ALT), and total bilirubin (TBIL).

Subsequent to blood collection, mice were euthanized, and major organs (heart, liver, spleen, lungs, and kidneys) were promptly harvested. Tissues were fixed in 4% paraformaldehyde for 24 h, routinely paraffin-embedded, sectioned into 5-μm slices, and stained with H&E. Stained sections were examined under a light microscope to assess pathological changes such as inflammatory infiltration, cellular necrosis, and structural abnormalities across all groups.

### Statistical analysis

All statistical analyses were performed using GraphPad Prism 10.0 and IBM SPSS Statistics v.23.0. Quantitative data are presented as mean ± standard deviation (SD), with sample sizes specified for each experiment. For comparisons between 2 independent groups, unpaired 2-tailed Student *t* test was used. For multiple group comparisons, one-way analysis of variance (ANOVA) followed by Tukey’s post hoc test was applied. Statistical significance was defined as *P* < 0.05, with different levels denoted as *P < 0.05, **P < 0.01, ***P < 0.001, and ****P < 0.0001. “ns” indicates no significant difference (*P* ≥ 0.05). Each experiment was independently repeated at least 3 times.

## Results and Discussion

### Synthesis and characterization

**Fig. 1.**
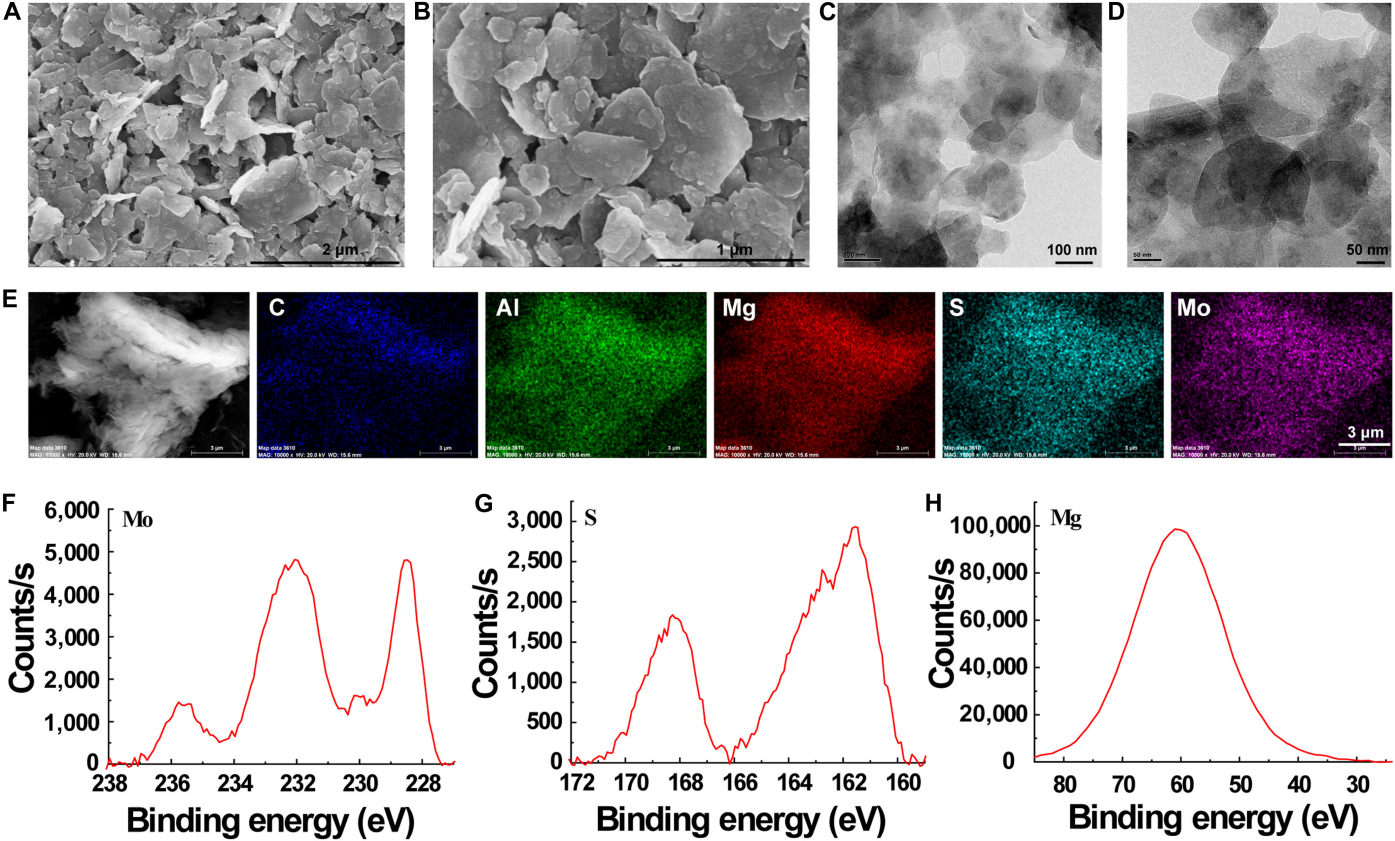
Synthesis and characterization of the LM@BSA nanocomposite. (A and B) Scanning electron microscopy (SEM) of LM@BSA nanocomposite. (C and D) Transmission electron microscopy (TEM) of LM@BSA nanocomposite. (E) Elemental distribution of LM@BSA nanocomposite. X-ray photoelectron spectroscopy (XPS) spectra of (F) Mo, (G) S, and (H) Mg of LM@BSA nanocomposite.

LM nanosheets were prepared via a hydrothermal method. Subsequently, the LM nanosheets were coated with BSA through electrostatic interactions to form the LM@BSA nanocomposite. As revealed by SEM (Fig. 1A and B) and TEM (Fig. 1C and D) analyses, the LM@BSA nanocomposite exhibited a unique sheet-like morphology. The diameter of the nanocomposite was approximately 0.637 μm. The microscopic morphology of LM@BSA was further characterized by atomic force microscopy. A substantial amount of molybdenum sulfide was deposited on the surface of the LDH nanosheets, presenting distinct surface undulations and coverage features with a maximum height peak of approximately 284 nm (Fig. S1). Furthermore, elemental mapping (Fig. 1E) confirms the uniform distribution of Mg, Al, Mo, and S elements throughout the LM@BSA nanocomposite, confirming the uniform composition of the nanocomposite. To further elucidate the surface chemical composition and valence states, XPS analysis was performed. The XPS spectrum of Mo (Fig. 1F) displays characteristic peaks located at 232.0 and 228.5 eV, which can be attributed to Mo^4+^ 3d. Meanwhile, S^2−^ peaks at 161.8 eV (2p1/2) and 168.4 eV (2p3/2) have also been observed (Fig. 1G), indicating that MoS_2_ were successfully prepared. Additionally, the Mg 1s spectrum (Fig. 1H) exhibits a strong signal in the 60.2 eV, verifying the existence of Mg, which originates from the LDH nanosheets.

**Fig. 2.**
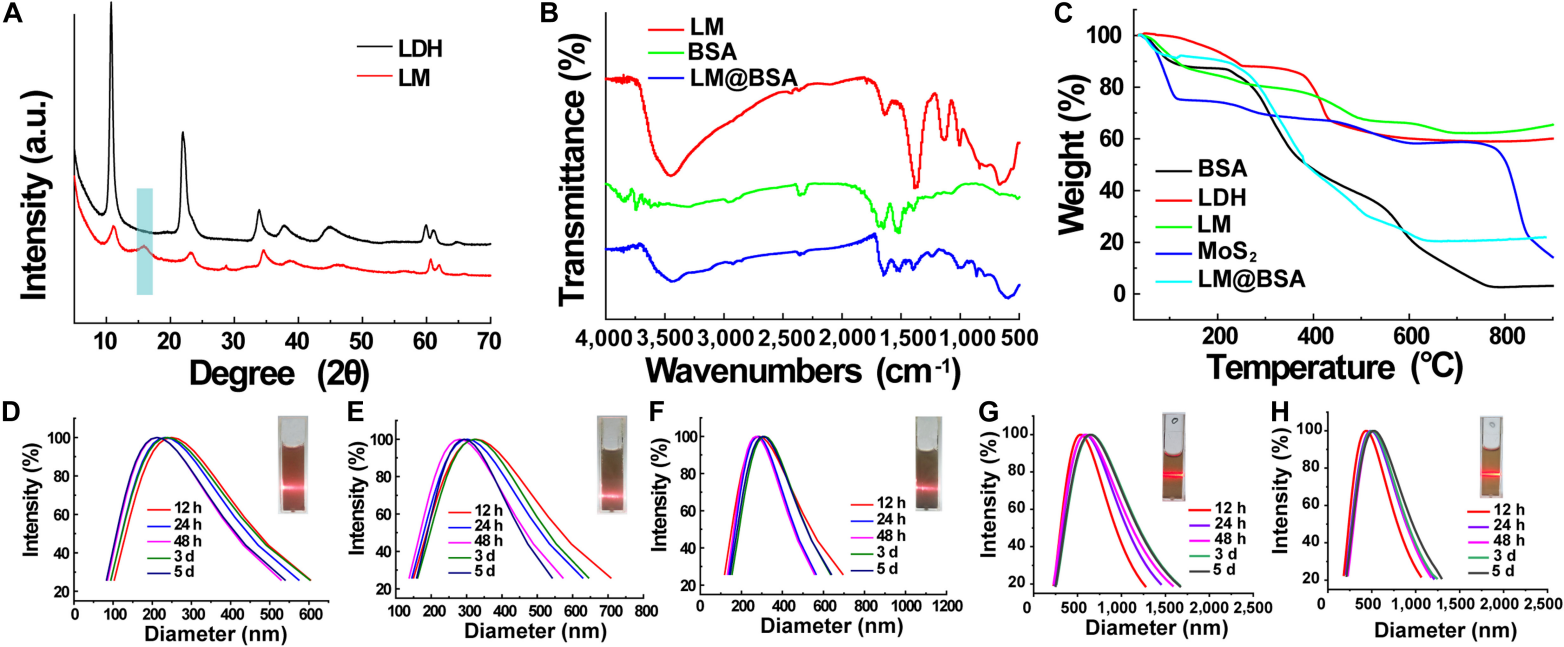
Optical properties and photothermal performance of LM@BSA nanocomposite. (A) X-ray diffraction (XRD) spectra of layered double hydroxide (LDH) nanosheets and LM nanosheets. (B) Fourier transform infrared (FTIR) spectrum of BSA, LM nanosheets, and LM@BSA nanocomposite. (C) The thermogravimetric analysis (TGA) curve of the nanocomposite and its individual components. Dynamic light scattering (DLS) and photographs of LM@BSA nanocomposite in (D) Dulbecco’s modified Eagle medium (DMEM), (E) saline, (F) deionized (DI) water, (G) simulated gastric fluid (SGF), and (H) simulated intestinal fluid (SIF), respectively. The corresponding DLS profiles of LM nanosheets under the same conditions are shown in Fig. S2.

The crystal structures of LDH nanosheets and LM nanosheets were analyzed by XRD. The diffractograms show the presence of low-intensity but recognizable diffraction features of LM nanosheets around 2θ ≈ 15°, corresponding to the (002) crystal planes of MoS_2_ (PDF#37-1492), confirming the successful formation of LM nanosheets (Fig. 2A). Further verification of the surface BSA modification was provided by FTIR spectroscopy. A strong absorption peak was observed at 1,532 cm^−1^, which corresponds to the -CO-NH- in BSA. The same absorption peak was observed in the LM@BSA nanocomposite, indicating the successful coating of BSA onto the LM nanosheets (Fig. 2B). The thermal behavior was assessed by thermogravimetric analysis (TGA). Pure BSA exhibited significant weight loss, attributed to pyrolysis of its protein backbone. In contrast, the LM nanosheets and LDH nanosheets demonstrated superior thermal stability, exhibiting slower and less pronounced mass loss across the entire temperature range. Notably, the LM@BSA nanocomposite displayed an intermediate thermal degradation profile, with a residual mass higher than that of BSA alone, attributable to the incorporation of thermally stable inorganic components (LDH and MoS_2_) from the LM core (Fig. 2C). Moreover, the LM@BSA nanocomposite exhibited a pronounced Tyndall effect in various media including DMEM, physiological saline, DI water, SGF, and SIF. The hydrodynamic diameter remained largely consistent over 3 days, with only minor variations, demonstrating excellent stability under physiologically relevant conditions (Fig. 2D to H), whereas the corresponding DLS profiles of LM nanosheets under the same conditions showed less stable dispersion behavior (Fig. S2).

### In vitro biocompatibility and ROS-scavenging capacity

The biocompatibility of the LM@BSA nanocomposite was first assessed using mouse fibroblasts (L929). The results of the CCK-8 assay indicated that the LM@BSA nanocomposite, at concentrations ranging from 0 to 100 μg/ml, exhibited minimal cytotoxicity (Fig. S3). This promising biocompatibility was further confirmed by Live/Dead staining using Calcein-AM/PI. Figure S4 illustrates strong green fluorescence from viable cells and very few red signals from dead cells across all treatment groups, collectively demonstrating the high biocompatibility of LM@BSA nanocomposite, making it suitable for further biological investigations.

**Fig. 3.**
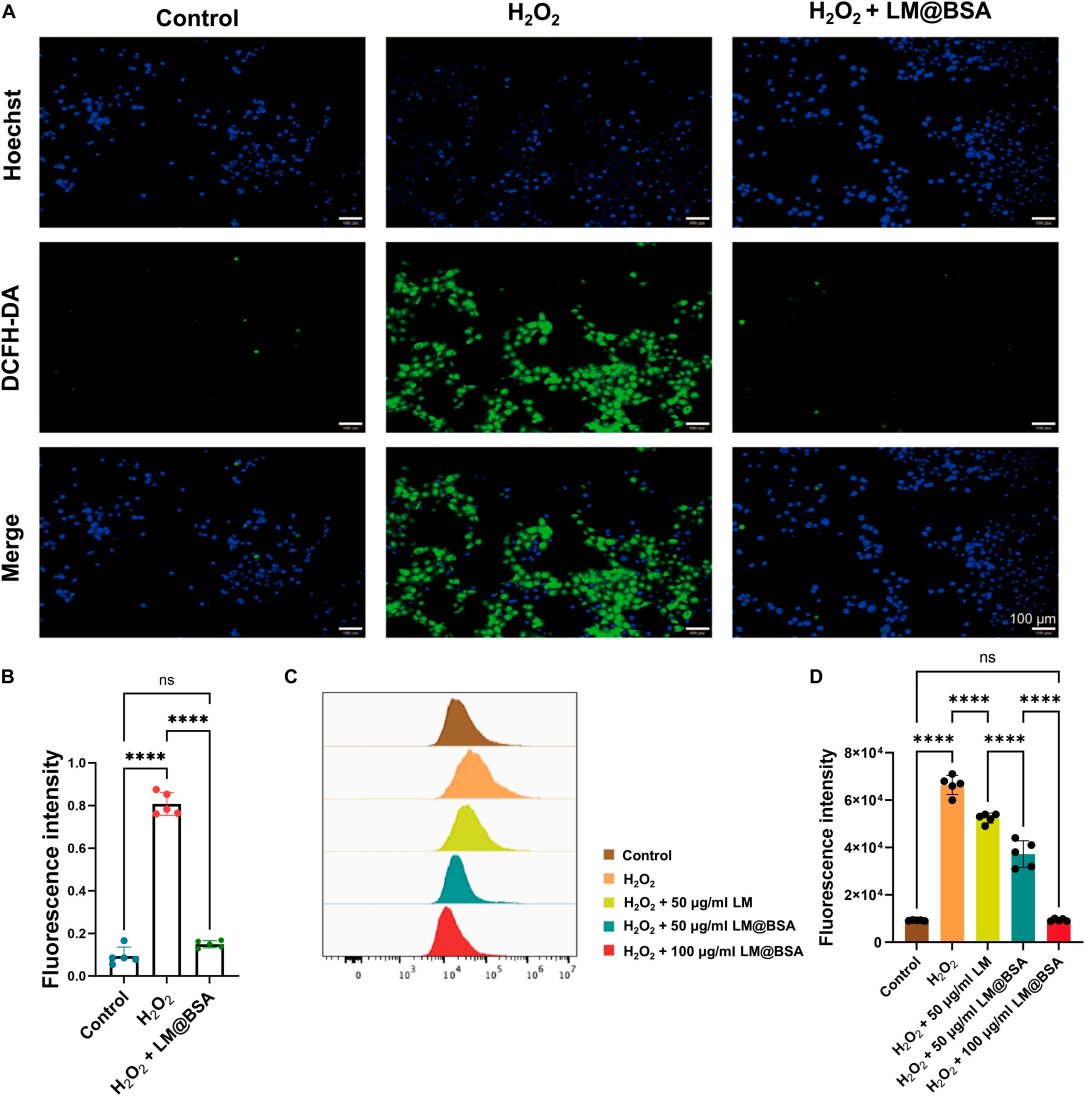
In vitro biocompatibility and reactive oxygen species (ROS)-scavenging capacity. (A) Fluorescence microscopy images of intracellular ROS levels under different conditions. (B) Quantitative analysis of ROS fluorescence intensity. (C and D) Flow cytometry analysis and quantification of intracellular ROS levels. Data are presented as mean ± SD. Statistical significance was determined by one-way analysis of variance (ANOVA) followed by Tukey’s post hoc test. ****P < 0.0001, ns: not significant.

The ROS-scavenging capacity of LM@BSA nanocomposite was studied under oxidative stress induced by hydrogen peroxide (H_2_O_2_). To simulate ROS-mediated oxidative stress, RAW264.7 cells were exposed to 100 mM H_2_O_2_ and subsequently coincubated with the LM@BSA nanocomposite. Fluorescence microscopy images (Fig. 3A) were captured using the DCFH-DA and DAPI for nuclear staining. The control group displayed intact cell morphology, weak DCFH-DA fluorescence indicating low intracellular ROS, and uniform nuclear staining. In contrast, the H_2_O_2_ group exhibited significant morphological damage, intense green fluorescence suggesting substantial ROS buildup, and disorganized nuclei. Remarkably, the H_2_O_2_ + LM@BSA nanocomposite group showed notably reduced DCFH-DA fluorescence, restored cell morphology, and normal nuclear configuration similar to the control group. Quantitative analysis of fluorescence intensity further corroborated the findings, showing that ROS levels in the LM@BSA nanocomposite-treated group were significantly lower than those in the H_2_O_2_-only group (Fig. 3B). These results confirm that the LM@BSA nanocomposite effectively scavenges ROS and protects cells from oxidative damage, underscoring the potential of the LM@BSA nanocomposite in mitigating oxidative stress and preserving cellular health.

### Intracellular ROS elimination and mitochondrial integrity preservation

**Fig. 4.**
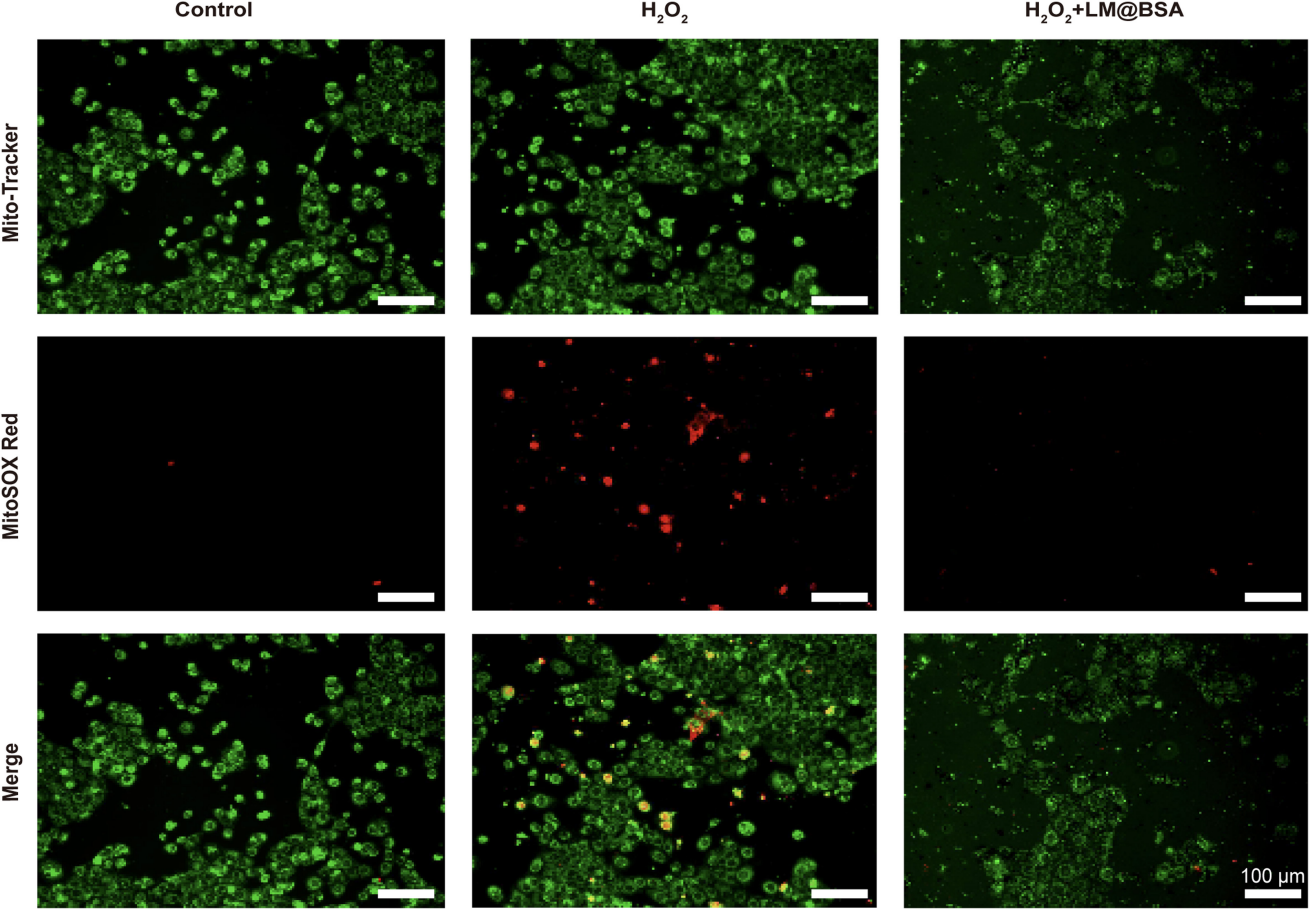
In vitro mitochondrial ROS-scavenging capacity. Fluorescence colocalization imaging of mitochondria (green) and mitochondrial ROS (red).

To elucidate the mechanisms behind LM@BSA nanocomposite’s antioxidant effects, we assessed total intracellular ROS, mitochondrial superoxide levels, and mitochondrial morphology in NCM-460 cells under oxidative stress induced by H_2_O_2_. Flow cytometry analysis (Fig. 3C and D) revealed that the H_2_O_2_ group exhibited a rightward shift in the fluorescence peak, indicating an increase in total ROS levels. In contrast, cells treated with the LM@BSA nanocomposite demonstrated leftward peak shifts, with the LM@BSA nanocomposite group’s profile closely aligning with the control group, suggesting effective ROS reduction. The stronger ROS-reducing effect of LM@BSA relative to uncoated LM may be attributed to the improved colloidal dispersion and interfacial biocompatibility conferred by the BSA shell. This coating likely facilitates more effective contact with cells, thereby enhancing its antioxidant activity. To specifically examine mitochondrial integrity, cells were costained with Mito-Tracker Green, reflecting mitochondrial morphology, and MitoSOX Red, indicating mitochondrial superoxide presence. As illustrated in Fig. 4, the control group displayed intact mitochondrial networks with minimal MitoSOX Red fluorescence, indicating low superoxide levels. Conversely, the H_2_O_2_ group exhibited fragmented mitochondria with disrupted green staining and intense red MitoSOX fluorescence. Merged images indicated extensive yellow-orange overlap, consistent with superoxide accumulation in damaged mitochondria. Notably, the group treated with H_2_O_2_ and 100 μg/ml LM@BSA nanocomposite displayed restored mitochondrial networks, significantly reduced MitoSOX Red fluorescence, and minimal yellow-orange colocalization in merged images. These findings confirm that the LM@BSA nanocomposite not only reduces total intracellular ROS in a dose-dependent manner but also specifically targets mitochondrial superoxide, preserving mitochondrial structure. The localization to mitochondria and capability to mitigate superoxide underscore LM@BSA nanocomposite’s dual action in mitigating oxidative damage, highlighting its multifaceted role in alleviating oxidative stress-induced cellular damage.

### Barrier repair and epithelial regeneration in intestinal organoids

**Fig. 5.**
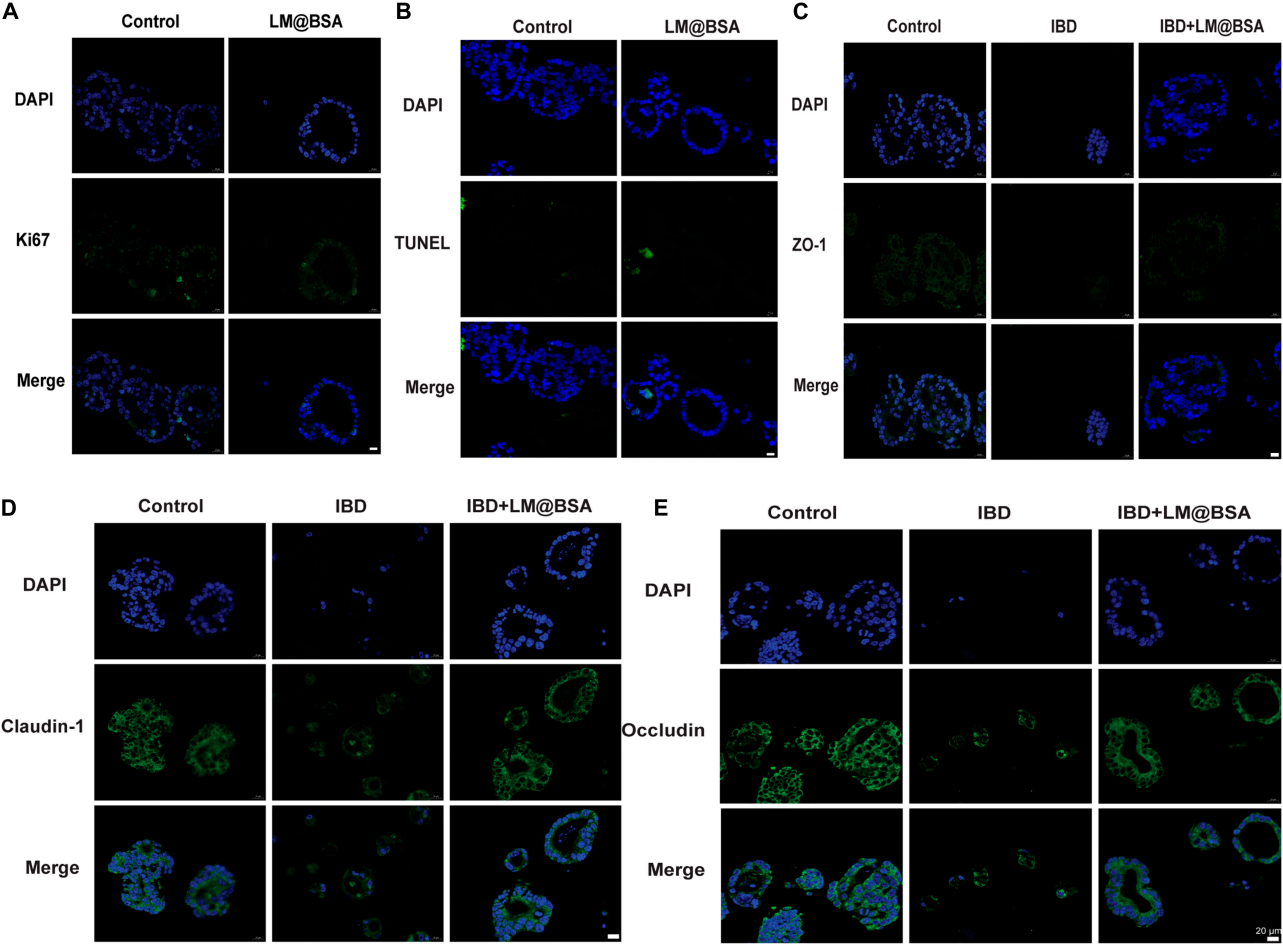
LM@BSA nanocomposite promotes epithelial regeneration and enhances barrier integrity in intestinal organoids. Immunofluorescence staining of (A) cell proliferation marker Ki-67, (B) apoptosis marker terminal deoxynucleotidyl transferase-mediated dUTP nick end labeling (TUNEL), and (C to E) tight junction proteins ZO-1, Claudin-1, and Occludin.

To investigate the biological effects of the LM@BSA nanocomposite on the intestinal epithelium, we first evaluated its impact on epithelial cell turnover under normal culture conditions. Mouse colon organoids were cultured in Matrigel and assigned to 2 groups: untreated controls (Control group) and LM@BSA-treated organoids (LM@BSA group). Immunofluorescence staining for Ki-67, a nuclear proliferation marker, revealed comparable numbers and distribution of positive nuclei between the 2 groups, indicating that the LM@BSA nanocomposite did not significantly alter basal proliferative activity (Fig. 5A). Similarly, apoptosis analysis using the TUNEL assay showed no appreciable difference in the frequency or intensity of TUNEL-positive cells between LM@BSA-treated and control organoids (Fig. 5B), suggesting that LM@BSA nanocomposite does not affect basal apoptotic rates in healthy epithelium.

We next examined the reparative potential of LM@BSA nanocomposite in an inflammatory injury model. Intestinal barrier damage was induced by treating colon organoids with TNF-α (50 ng/ml) for 24 h. After injury induction, organoids were maintained in either normal medium (IBD group) or medium supplemented with the LM@BSA nanocomposite (IBD + LM@BSA group) for 5 days, with untreated organoids serving as healthy controls (Control group). Immunofluorescence staining for key tight junction proteins including ZO-1 (Fig. 5C), Claudin-1 (Fig. 5D), and Occludin (Fig. 5E) revealed markedly reduced fluorescence intensity and disrupted, discontinuous localization along cell borders in the IBD group, consistent with severe barrier impairment. LM@BSA nanocomposite treatment substantially restored both expression and junctional organization of these proteins, yielding staining patterns and signal intensities comparable to those in the control group. This restoration suggests that LM@BSA effectively counteracts TNF-α-induced barrier disruption. Taken together, these results indicate that the LM@BSA nanocomposite does not alter basal epithelial proliferation or apoptosis under physiological conditions, while exerting significant reparative effects on tight junction integrity in the context of inflammatory injury.

### Suppression of intestinal inflammation and tissue protection in IBD mice

**Fig. 6.**
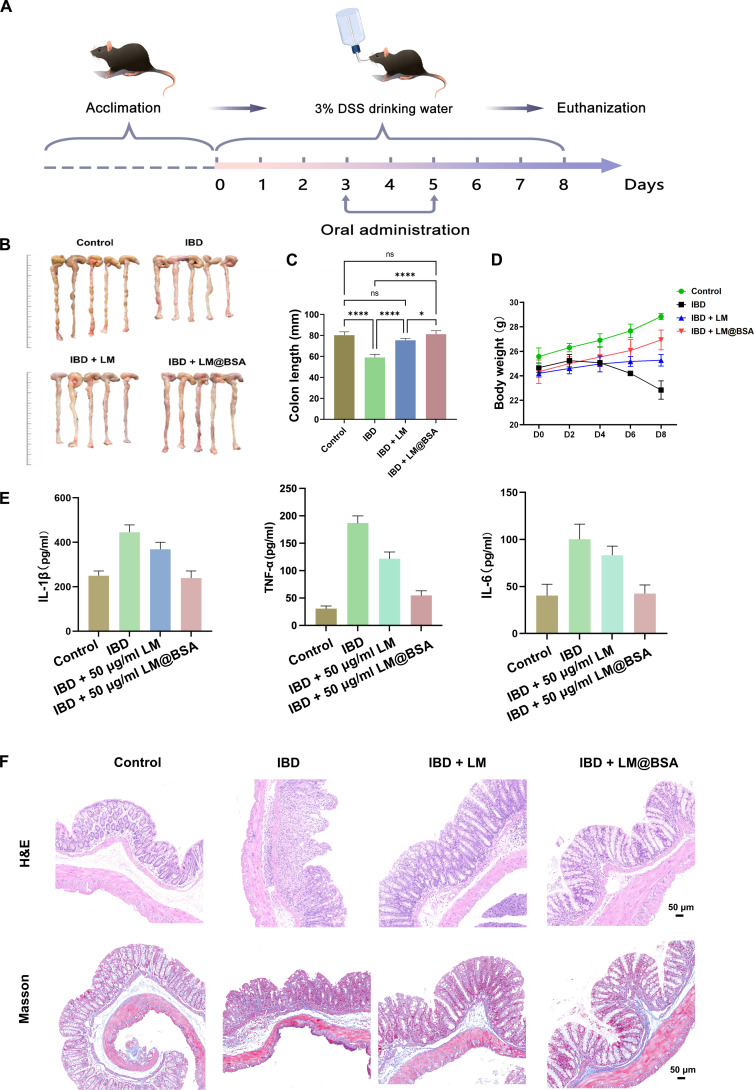
LM@BSA nanocomposite ameliorates IBD in mice. (A) Schematic diagram of the experimental timeline for IBD and LM@BSA nanocomposite treatment. (B and C) Representative images and quantitative analysis of colon length. (D) Body weight changes of mice throughout the experimental period. (E) Levels of pro-inflammatory cytokines (IL-1β, TNF-α, and IL-6) in colon tissues. (F) Representative hematoxylin and eosin (H&E) staining and Masson’s trichrome staining showing collagen deposition. Higher-magnification images are provided in Fig. S5. Data are presented as mean ± SD. Statistical significance was determined by one-way ANOVA followed by Tukey’s post hoc test. Significance levels are denoted as *P < 0.05, **P < 0.01, ***P < 0.001, and ****P < 0.0001; ns, not significant.

Building on LM@BSA nanocomposite’s strong ROS-scavenging capabilities and colloidal stability, we assessed its in vivo therapeutic potential using a DSS-induced IBD model in C57BL/6 mice. The experimental design is illustrated in Fig. 6A. The LM@BSA nanocomposite was orally administered on days 3 and 5, followed by euthanasia for tissue analysis. Colon length served as a readout of disease severity, and LM@BSA nanocomposite treatment markedly alleviated DSS-induced colon shortening (Fig. 6B and C), indicating protection against colonic injury. Body weight changes further supported this therapeutic effect: mice in the IBD group showed marked body weight loss, whereas healthy control mice gained weight. In contrast, mice treated with LM@BSA nanocomposites maintained stable body weight after 2 oral administrations (on days 3 and 5) and showed a clear trend toward recovery by the experimental endpoint. Mice treated with uncoated LM also showed stabilization of body weight, though to a lesser extent than the LM@BSA group. These results indicate that LM@BSA nanocomposite treatment effectively alleviated the systemic effects of disease progression (Fig. 6D).

These marked improvements in disease phenotypes prompted us to investigate the underlying molecular mechanisms. We hypothesized that the ROS-scavenging activity of LM@BSA would lead to a reduction in downstream inflammatory signaling. Therefore, we quantified pro-inflammatory cytokines (IL-1β, TNF-α, and IL-6) in colon tissues using ELISA. As depicted in Fig. 6E, LM@BSA nanocomposite treatment significantly reduced pro-inflammatory cytokine levels: IL-1β and TNF-α were markedly down-regulated, and IL-6 levels were substantially decreased compared to the IBD group. This reduction in cytokine production underscores LM@BSA nanocomposite’s capability to alleviate intestinal inflammation, confirming that the alleviation of clinical symptoms is directly associated with the modulation of the local inflammatory milieu by LM@BSA. Further supporting these results, histological analyses using H&E and Masson’s staining revealed extensive inflammatory cell infiltration in the intestines of IBD mice, which was significantly reduced in the LM@BSA nanocomposite-treated group (Fig. 6F and Fig. S5). Collectively, these data support the therapeutic potential of LM@BSA in mitigating DSS-induced colonic inflammation and tissue damage.

### Inhibition of ferroptosis in IBD mice

**Fig. 7.**
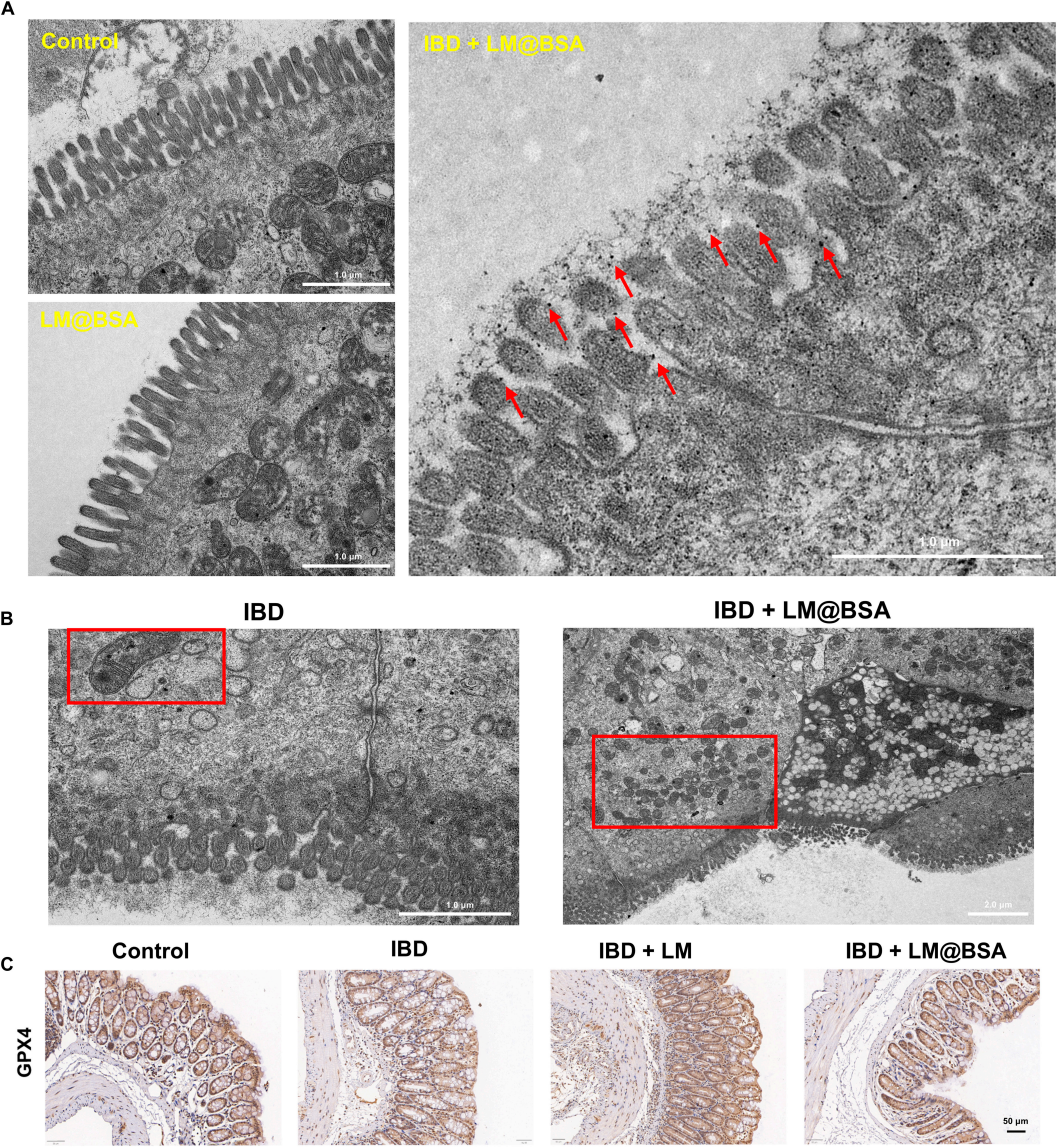
LM@BSA nanocomposite alleviates colitis by inhibiting ferroptosis. (A) Bio-TEM images of LM@BSA nanocomposite in colonic tissue (red arrows). (B) Ultrastructural analysis of colonic epithelial cells by TEM. (C) Immunohistochemical staining of GPX4 in colonic tissue. Quantitative analysis is provided in Fig. S6.

To elucidate the protective mechanisms and in vivo behavior of the LM@BSA nanocomposite, we conducted bio-TEM and immunohistochemical analyses on distal colon tissues. First, we assessed the LM@BSA nanocomposite’s biocompatibility and lesion-associated localization using bio-TEM (Fig. 7A). A comparison between healthy control mice (Control group) and healthy mice administered the LM@BSA nanocomposite (LM@BSA group) revealed little histological difference, with both groups showing intact intestinal architecture and normal villus morphology. This confirms that the LM@BSA nanocomposite does not disrupt intestinal homeostasis in healthy tissue. In the IBD + LM@BSA group, the nanocomposite was observed adjacent to injured mucosal regions (indicated by red arrows), suggesting lesion-associated localization near inflamed colonic tissue. This distribution pattern suggests that the BSA coating may contribute to local retention of the nanocomposite within the injured mucosal microenvironment, potentially through enhanced interfacial stability and mucosal interaction. Such an interpretation is consistent with previous reports showing that albumin-based systems possess favorable biophysical and drug-delivery properties [39], that surface-engineered albumin nanocarriers can enhance binding and retention in inflamed intestinal tissue [40], and that BSA-containing oral nanosystems may improve prolonged residence in inflamed colonic regions [41]. However, the precise role of the BSA shell in this process remains to be further clarified in future studies.

At the ultrastructural level, bio-TEM analysis further provided morphological evidence consistent with attenuation of ferroptosis-related injury (Fig. 7B). Colon epithelial cells from the IBD group displayed typical mitochondrial alterations associated with ferroptosis, including diminished cristae and increased membrane density. In contrast, mitochondria from the LM@BSA nanocomposite-treated group retained a normal morphology with well-defined cristae, indicating that the LM@BSA nanocomposite effectively protects mitochondrial integrity against ferroptosis-associated damage. The observed preservation of mitochondrial structure suggested a protective effect against ferroptosis-associated injury. To further evaluate ferroptosis-related molecular changes, we evaluated the expression of the key ferroptosis regulator, GPX4, via immunohistochemistry (Fig. 7C). As expected, the IBD group exhibited significantly weaker GPX4 staining compared to the control group, supporting the presence of ferroptosis-related injury in colonic tissue. Quantitative analysis of GPX4 staining intensity further confirmed this observation, showing that the mean gray value in the IBD group was significantly higher than that of the control group, whereas both LM and LM@BSA treatments markedly reduced this value (Fig. S6). Notably, no statistically significant difference was observed between the LM and LM@BSA groups. Taken together with the preservation of mitochondrial ultrastructure, these findings support a coordinated anti-ferroptotic protective effect of LM@BSA in DSS-induced colitis. Collectively, the protection of mitochondrial ultrastructure and the recovered GPX4 staining pattern provide a coherent basis to support that LM@BSA scavenges ROS, thereby mitigating ferroptosis-associated epithelial injury and protecting the epithelial barrier.

### Transcriptomic orchestration of tissue remodeling and immune balance

**Fig. 8.**
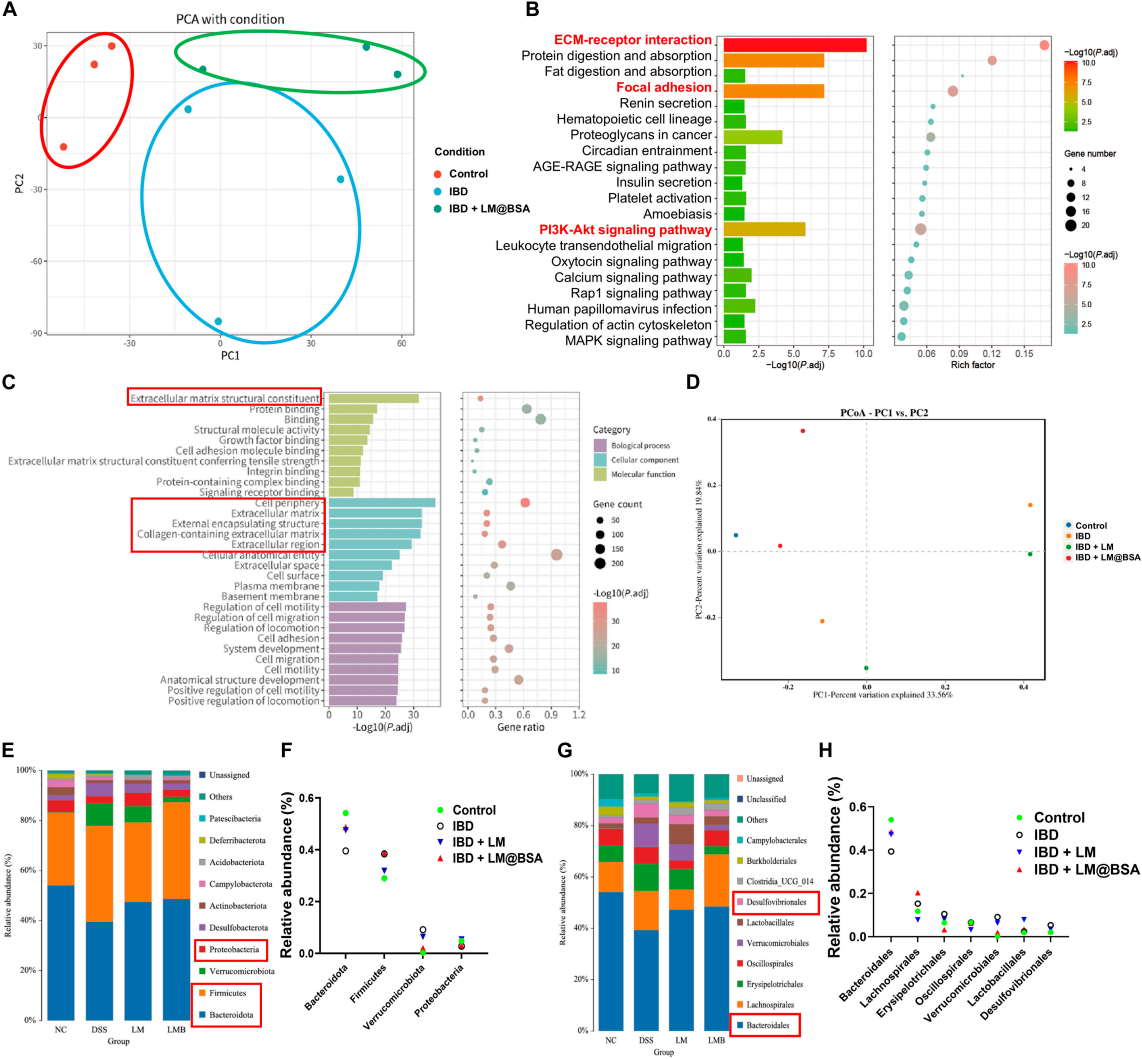
Multiomics analyses reveal that LM@BSA nanocomposite restores gut homeostasis by coordinating transcriptomic reprogramming and microbial restructuring. (A) Principal component analysis (PCA) of colonic transcriptomes. (B) Kyoto Encyclopedia of Genes and Genomes (KEGG) pathway enrichment analysis. (C) Gene Ontology (GO) pathway enrichment analysis. (D) PCA based on Bray–Curtis dissimilarity. (E and G) Stacked bar plots display the relative abundance of bacterial communities at the phylum and order levels. (F and H) Dynamic profiles of significantly altered bacterial phyla and specific bacterial orders across different experimental groups.

To gain a comprehensive understanding of the therapeutic mechanisms of LM@BSA nanocomposite beyond ferroptosis inhibition, transcriptomic analyses were conducted. The results revealed significant insights into the molecular mechanisms underlying its effects. Firstly, principal component analysis (PCA) revealed that the overall characteristics of the samples in the Control, IBD, and IBD + LM@BSA nanocomposite-treated groups were significantly different, roughly forming relatively independent aggregation regions (Fig. 8A). Transcriptome profiling highlighted an enrichment of terms related to the extracellular matrix (ECM) and cell adhesion, particularly in ECM–receptor interaction and focal adhesion pathways. These enrichments, driven by down-regulated genes in the LM@BSA nanocomposite-treated group (Fig. 8B), suggest that the LM@BSA nanocomposite effectively regulates ECM dynamics. This regulation appears to curtail excessive pathological ECM remodeling, such as fibrosis, caused by the inflammatory microenvironment in IBD. Concurrently, the up-regulation of Gene Ontology (GO) terms concerning ECM structural components indicates that the LM@BSA nanocomposite promotes ECM synthesis for tissue repair, providing a scaffold necessary for effective tissue recovery (Fig. 8C). This transcriptional reprogramming toward balanced tissue repair is likely a downstream consequence of the reduced oxidative stress and cellular damage (ferroptosis inhibition) mediated by LM@BSA. By mitigating the primary insults, the nanocomposite creates a tissue environment conducive to orderly healing rather than destructive remodeling. This interpretation is supported by histological analysis, which shows that LM@BSA nanocomposite treatment significantly reduced collagen deposition and improved tissue structure, as illustrated by Masson’s staining (Fig. 6F).

Secondly, the analysis revealed significant enrichment of immune-related pathways, including antigen processing and presentation, nature killer cell-mediated cytotoxicity, and T helper 1 (Th1)/T helper 2 (Th2) cell differentiation (Fig. S7). This enrichment signifies a critical systemic immune adaptation that emerges as a downstream outcome of the primary cellular protection conferred by LM@BSA. In the context of DSS-induced colitis, characterized by immune imbalance, the transcriptional patterns observed with LM@BSA nanocomposite treatment suggest normalization of immune function rather than mere pro-inflammatory activation. Mechanistically, by reducing oxidative stress and mitigating ferroptosis, LM@BSA attenuates the persistent damage signals that otherwise drive chronic immune activation. This creates a tissue environment that allows the immune system to rebalance, thus enhancing antigen presentation and cytotoxic activity. This likely facilitates more effective clearance of damaged cells, transitioning from a state of disordered inflammation to a coordinated resolving immune response. Consequently, the transcriptomic shift toward immune coordination is a direct result of the ameliorated tissue microenvironment, effectively bridging the gap between cytoprotection and systemic immune modulation. The significant decrease in pro-inflammatory cytokines (TNF-α, IL-1β, and IL-6) in LM@BSA nanocomposite-treated tissues provides a functional protein-level confirmation of this transcriptional rebalancing effect.

Additionally, an intriguing finding was the significant enrichment of the phosphoinositide 3-kinase–protein kinase B signaling pathway, predominantly seen in the down-regulated gene cluster (Fig. 8B). Given its central role in regulating cell survival, metabolism, and inflammation, this modulation is of considerable interest. In DSS-induced hyper-inflammatory conditions, the pathway’s overactivation contributes to disease progression by promoting inflammatory signaling and dysregulated cell survival. The ability of LM@BSA to suppress this overactivation likely stems from its interruption of upstream triggers, such as oxidative stress and inflammatory cytokine release. Consequently, the down-regulation of PI3K–Akt signaling by the LM@BSA nanocomposite suggests a pivotal therapeutic mechanism through which it alleviates inflammation and aids in restoring intestinal homeostasis, potentially regulating pathological processes like excessive ECM remodeling and immune imbalances.

### Gut microbiota modulation in IBD mice

Principal coordinates analysis, based on Bray–Curtis (explaining 33.56% of variance) and Binary–Jaccard metrics (explaining 18.91% of variance), revealed a distinct separation between the IBD group (IBD) and the control group (Control), confirming that DSS administration significantly disrupted the gut microbial community (Fig. 8D and Fig. S8). Notably, the microbial communities of both the LM-treated (IBD + LM) and the more pronounced LM@BSA nanocomposite-treated groups (IBD + LM@BSA) demonstrated a significant shift from the IBD group cluster toward that of the control group, suggesting a restorative effect on gut dysbiosis induced by IBD. This restoration was quantified using alpha diversity indices, where IBD markedly reduced both Simpson and Shannon indices. In contrast, LM@BSA nanocomposite treatment restored microbial diversity to levels comparable to or exceeding those of the control group (Simpson: 0.981; Shannon: 7.317), with LM treatment also showing substantial improvement (Shannon: 7.1881, as shown in Fig. S9). The recovery of these alpha diversity indices not only signifies a quantitative restoration of species richness and evenness but also points toward an increased ecological stability and functional resilience of the gut ecosystem, which is often compromised in IBD.

At the phylum level, the IBD group was characterized by significant dysbiosis (Fig. 8E and F). Specifically, it exhibited a typical imbalance, with decreased abundance of the beneficial *Bacteroidota* phylum, which is prevalent in the control group, and increased abundance of *Firmicutes*, a recognized marker of gut inflammation. This shift in the *Firmicutes/Bacteroidota* ratio is a well-recognized signature of gut inflammation [42,43]. Ecologically, the rebalancing of this ratio by LM@BSA treatment indicates a fundamental shift from a dysbiotic state associated with inflammation back toward a homeostatic community structure, which is critical for maintaining normal gut function. Additionally, there was a decrease in *Proteobacteria*. Further analysis at the order level identified key taxonomic shifts contributing to these restorative effects (Fig. 8G and H). *Bacteroidales*, a major and stable component of the human gut microbiome involved in carbohydrate metabolism and nutrient supply [44,45], were significantly reduced in the IBD group, contributing to compromised metabolic functions and worsened dysbiosis. The recovery of *Bacteroidales* is crucial as members of this order are primary producers of short-chain fatty acids, such as butyrate, which serve as the main energy source for colonocytes and possess potent anti-inflammatory properties [44,46]. Remarkably, LM@BSA nanocomposite treatment promoted the recovery of *Bacteroidales* abundance to near control group levels, reflecting a re-establishment of beneficial metabolic capacities. Conversely, the abundance of *Desulfovibrionales*, an order of sulfate-reducing bacteria that produce hydrogen sulfide [47] and potentially exacerbate inflammation, was significantly increased in the IBD group. The hydrogen sulfide produced by *Desulfovibrionales* can impair colonocyte metabolism and weaken the mucosal barrier, directly linking this bacterial group to disease pathogenesis [48,49]. Crucially, the LM@BSA nanocomposite effectively suppressed *Desulfovibrionales* overgrowth, bringing its abundance closer to that of the control group. This reduction is likely pivotal in breaking the “microbiota–inflammation” cycle, thereby promoting mucosal healing and intestinal health.

### In vivo biocompatibility and biosafety profiles

To systematically assess in vivo biocompatibility, we performed H&E staining on heart, liver, spleen, lung, and kidney tissues (Fig. S10). The results showed that LM@BSA nanocomposite-treated mice had no obvious histological abnormalities such as inflammatory infiltration, necrosis, or structural disorder in any of the organs, which was very similar to the control group. Furthermore, we collected orbital blood from mice before euthanasia to measure serum markers of renal function (CREA and UREA) and hepatic function (AST, ALT, and TBIL). Serum levels of CREA, UREA, AST, ALT, and TBIL in the LM@BSA nanocomposite group (Fig. S11) remained within the physiological range, which was in sharp contrast to the significant increases observed in the IBD group. These findings support the favorable histocompatibility and biosafety of LM@BSA, which may reflect the inherent biocompatibility of its components together with its limited long-term tissue retention after oral administration. In addition, the contents of Al and Mo in major organs were examined by inductively coupled plasma mass spectrometry (ICP-MS). From the ICP-MS results (Fig. S12), it was evident that accumulation of Al and Mo was detected in the liver, kidneys, and colon at 24 h after administration, which is consistent with the physiological role of both during metabolism and excretion. By 72 h, there was a statistically significant decrease in all organs tested, none of which were statistically different from the control group, suggesting no significant long-term tissue accumulation, further confirming the favorable biosafety of LM@BSA for oral IBD treatment. Together with the high in vitro biocompatibility, these results indicate a favorable safety margin for LM@BSA.

Our study demonstrates that the biomimetic LM@BSA nanocomposite effectively treats IBD through a multimodal mechanism involving ROS scavenging, ferroptosis inhibition, immune modulation, and gut microbiota restoration, while offering superior safety, patient compliance, and local action over conventional IBD therapies. Compared with mainstream treatments such as injectable biologics, broad immunosuppressants, and antibiotics, LM@BSA avoids systemic immunosuppression, infection risks, and microbiota disruption, making it a highly favorable alternative. With further validation in large animal models, Good Manufacturing Practice-compliant scale-up, and long-term safety evaluation, LM@BSA holds great potential as a next-generation, microbiome-friendly nanotherapeutic for holistic intestinal health restoration in IBD.

## Conclusion

In summary, we developed a biomimetic LM@BSA nanocomposite with enzyme-like activity for the treatment of IBD through multidimensional synergistic effects. This formulation exhibited strong ROS-scavenging capacity, protected cells from oxidative injury, and mitigated ferroptosis-related damage by preserving mitochondrial integrity and restoring GPX4-associated changes. LM@BSA promoted intestinal barrier repair in vitro and was observed near injured mucosal regions in the DSS-induced colitis model, suggesting lesion-associated localization that may favor local retention and contribute to its therapeutic efficacy. Mechanistically, LM@BSA alleviated colonic inflammation through the regulation of pro-inflammatory cytokines, attenuation of ferroptosis-associated injury, transcriptomic reprogramming related to tissue remodeling and immune balance, and partial restoration of gut microbiota homeostasis. Furthermore, LM@BSA exhibited favorable biocompatibility and biosafety in vivo, supporting its translational potential as a promising nanotherapeutic strategy for IBD. By integrating ROS elimination, targeted delivery, barrier restoration, and immune regulation, the LM@BSA nanocomposite represents a promising therapeutic candidate for IBD and may also provide a reference strategy for the management of other ROS-mediated inflammatory disorders.

## Data Availability

The datasets used and/or analyzed during the current study are available from the corresponding authors upon reasonable request.

## References

[1] Torres J, Mehandru S, Colombel JF, Peyrin-Biroulet L. Crohn’s disease. Lancet. 2017;389(10080):1741–1755. 27914655 10.1016/S0140-6736(16)31711-1

[2] Qiao Y, Tang X, Liu Z, Wiredu Ocansey DK, Zhou M, Shang A, Mao F. Therapeutic prospects of mesenchymal stem cell and their derived exosomes in the regulation of the gut microbiota in inflammatory bowel disease. Pharmaceuticals. 2024;17(5):607. 38794176 10.3390/ph17050607 PMC11124012

[3] Xavier RJ, Podolsky DK. Unravelling the pathogenesis of inflammatory bowel disease. Nature. 2007;448(7152):427–434. 17653185 10.1038/nature06005

[4] Lloyd-Price J, Arze C, Ananthakrishnan AN, Schirmer M, Avila-Pacheco J, Poon TW, Andrews E, Ajami NJ, Bonham KS, Brislawn CJ, et al. Multi-omics of the gut microbial ecosystem in inflammatory bowel diseases. Nature. 2019;569(7758):655–662. 31142855 10.1038/s41586-019-1237-9 PMC6650278

[5] Nenci A, Becker C, Wullaert A, Gareus R, van Loo G, Danese S, Huth M, Nikolaev A, Neufert C, Madison B, et al. Epithelial NEMO links innate immunity to chronic intestinal inflammation. Nature. 2007;446(7135):557–561. 17361131 10.1038/nature05698

[6] Cao RY, Zhang Y, Feng Z, Liu S, Liu Y, Zheng H, Yang J. The effective role of natural product berberine in modulating oxidative stress and inflammation related atherosclerosis: Novel insights into the gut-heart axis evidenced by genetic sequencing analysis. Front Pharmacol. 2021;12: 764994. 35002703 10.3389/fphar.2021.764994 PMC8727899

[7] Zhang T, Gillies M, Wang Y, Shen W, Bahrami B, Zeng S, Zhu M, Yao W, Zhou F, Murray M, et al. Simvastatin protects photoreceptors from oxidative stress induced by all-trans-retinal, through the up-regulation of interphotoreceptor retinoid binding protein. Br J Pharmacol. 2019;176(12):2063–2078. 30825184 10.1111/bph.14650 PMC6534793

[8] Ru Q, Li Y, Chen L, Wu Y, Min J, Wang F. Iron homeostasis and ferroptosis in human diseases: Mechanisms and therapeutic prospects. Signal Transduct Target Ther. 2024;9(1):271. 39396974 10.1038/s41392-024-01969-z PMC11486532

[9] Huang W, Zhang Y, Das NK, Solanki S, Jain C, El-Derany MO, Koo I, Bell HN, Aabed N, Singhai R, et al. Fibroblast lipid metabolism through ACSL4 regulates epithelial sensitivity to ferroptosis in IBD. Nat Metab. 2025;7(7):1358–1374. 40571769 10.1038/s42255-025-01313-x PMC12444769

[10] M’koma AE. Inflammatory bowel disease: Clinical diagnosis and surgical treatment—Overview. Medicina. 2022;58(5):567. 35629984 10.3390/medicina58050567 PMC9144337

[11] Kushwaha P, Qureshi R, Khan N, Mukhopadhyay S. Revolutionizing IBD therapy: Insights into contemporary treatment strategies. Int Rev Immunol. 2025;45(1):1–25. 41027475 10.1080/08830185.2025.2563522

[12] Ghouri YA, Tahan V, Shen B. Secondary causes of inflammatory bowel diseases. World J Gastroenterol. 2020;26(28):3998–4017. 32821067 10.3748/wjg.v26.i28.3998 PMC7403802

[13] Colombel JF, Sandborn WJ, Reinisch W, Mantzaris GJ, Kornbluth A, Rachmilewitz D, Lichtiger S, D’Haens G, Diamond RH, Broussard DL, et al. Infliximab, azathioprine, or combination therapy for Crohn’s disease. N Engl J Med. 2010;362(15):1383–1395. 20393175 10.1056/NEJMoa0904492

[14] Feagan BG, Rutgeerts P, Sands BE, Hanauer S, Colombel J-F, Sandborn WJ, Assche GV, Axler J, Kim H-J, Danese S, et al. Vedolizumab as induction and maintenance therapy for ulcerative colitis. N Engl J Med. 2013;369(8):699–710. 23964932 10.1056/NEJMoa1215734

[15] Herrlinger KR, Stange EF. Twenty-five years of biologicals in IBD: What’s all the hype about? J Intern Med. 2021;290(4):806–825. 34128571 10.1111/joim.13345

[16] Kapizioni C, Desoki R, Lam D, Balendran K, Al-Sulais E, Subramanian S, Rimmer JE, Negro JDLR, Pavey H, Pele L, et al. Biologic therapy for inflammatory bowel disease: Real-world comparative effectiveness and impact of drug sequencing in 13 222 patients within the UK IBD BioResource. J Crohns Colitis. 2024;18(6):790–800. 38041850 10.1093/ecco-jcc/jjad203 PMC11147798

[17] Yaqin Z, Kehan W, Yi Z, Naijian W, Wi Q, Fei M. Resveratrol alleviates inflammatory bowel disease by inhibiting JAK2/STAT3 pathway activity via the reduction of O-GlcNAcylation of STAT3 in intestinal epithelial cells. Toxicol Appl Pharmacol. 2024;484: 116882. 38437956 10.1016/j.taap.2024.116882

[18] Paramsothy S, Kamm MA, Kaakoush NO, Walsh AJ, van den Bogaerde J, Samuel D, Leong RW, Connor S, Ng W, Paramsothy R, et al. Multidonor intensive faecal microbiota transplantation for active ulcerative colitis: A randomised placebo-controlled trial. Lancet. 2017;389(10075):1218–1228. 28214091 10.1016/S0140-6736(17)30182-4

[19] Sokol H, Landman C, Seksik P, Berard L, Montil M, Nion-Larmurier I, Bourrier A, Le Gall G, Lalande V, De Rougemont A, et al. Fecal microbiota transplantation to maintain remission in Crohn’s disease: A pilot randomized controlled study. Microbiome. 2020;8(1):12. 32014035 10.1186/s40168-020-0792-5 PMC6998149

[20] Yadegar A, Bar-Yoseph H, Monaghan TM, Pakpour S, Severino A, Kuijper EJ, Smits WK, Terveer EM, Neupane S, Nabavi-Rad A, et al. Fecal microbiota transplantation: Current challenges and future landscapes. Clin Microbiol Rev. 2024;37(2): e0006022. 38717124 10.1128/cmr.00060-22 PMC11325845

[21] Yasmin F, Najeeb H, Shaikh S, Hasanain M, Naeem U, Moeed A, Koritala T, Hasan S, Surani S. Novel drug delivery systems for inflammatory bowel disease. World J Gastroenterol. 2022;28(18):1922–1933. 35664964 10.3748/wjg.v28.i18.1922 PMC9150062

[22] Bao M, Wang K, Li J, Li Y, Zhu H, Lu M, Zhang Y, Fan Q, Han L, Wang K, et al. ROS scavenging and inflammation-directed polydopamine nanoparticles regulate gut immunity and flora therapy in inflammatory bowel disease. Acta Biomater. 2023;161:250–264. 36863680 10.1016/j.actbio.2023.02.026

[23] Gao J, Li J, Luo Z, Wang H, Ma Z. Nanoparticle-based drug delivery systems for inflammatory bowel disease treatment. Drug Des Devel Ther. 2024;18:2921–2949. 10.2147/DDDT.S461977 PMC11269238 39055164

[24] Xiang S, Zhan H, Zhan J, Li X, Lin X, Sun W. Breaking hypoxic barrier: Oxygen-supplied nanomaterials for enhanced T cell-mediated tumor immunotherapy. Int J Pharm X. 2025;10: 100400. 41048631 10.1016/j.ijpx.2025.100400 PMC12495343

[25] Zhang C, Wang H, Yang X, Fu Z, Ji X, Shi Y, Zhong J, Hu W, Ye Y, Wang Z, et al. Oral zero-valent-molybdenum nanodots for inflammatory bowel disease therapy. Sci Adv. 2022;8(37):eabp9882. 36112678 10.1126/sciadv.abp9882 PMC9481133

[26] Ni D, Jiang D, Kutyreff CJ, Lai J, Yan Y, Barnhart TE, Yu B, Im H-J, Kang L, Cho SY, et al. Molybdenum-based nanoclusters act as antioxidants and ameliorate acute kidney injury in mice. Nat Commun. 2018;9(1):5421. 30575745 10.1038/s41467-018-07890-8 PMC6303396

[27] Constantino VRL, Figueiredo MP, Magri VR, Eulálio D, Rodrigues Cunha VR, Santos Alcântara AC, Perotti GF, et al. Biomaterials based on organic polymers and layered double hydroxides nanocomposites: Drug delivery and tissue engineering. Pharmaceutics. 2023;15(2):413. 36839735 10.3390/pharmaceutics15020413 PMC9961265

[28] Zhao J, Wu H, Zhao J, Yin Y, Zhang Z, Wang S, Lin K. 2D LDH-MoS_2_ clay nanosheets: Synthesis, catalase-mimic capacity, and imaging-guided tumor photo-therapy. J Nanobiotechnol. 2021;19(1):36. 10.1186/s12951-020-00763-7 PMC7860036 33536031

[29] Cui J, Zhang L, Li J, Chen Y, Dai S, Maitz MF, Zhao A, Yang P. Layered double hydroxides-based nanozymes for effective biomedical applications: A review and future perspectives. BMEMat. 2025;4(1): e70013.

[30] Zhang D, Cui P, Dai Z, Yang B, Yao X, Liu Q, Hu Z, Zheng X. Tumor microenvironment responsive FePt/MoS_2_ nanocomposites with chemotherapy and photothermal therapy for enhancing cancer immunotherapy. Nanoscale. 2019;11(42):19912–19922. 31599915 10.1039/c9nr05684j

[31] Yu P, Li Y, Sun H, Zhang H, Kang H, Wang P, Xin Q, Ding C, Xie J, Li J. Mimicking antioxidases and hyaluronan synthase: A zwitterionic nanozyme for photothermal therapy of osteoarthritis. Adv Mater. 2023;35(44): e2303299. 37459592 10.1002/adma.202303299

[32] Wang T, Zhang F, Guimarães CF, Reis RL, Lv Y, Qu Y, Liu D, Zhou Q, Kong X, Shi J. Zn/Cu bi-single-atom nanoplatform: Hexagonal anti-tumor warrior by ROS amplification for DOX resistance reversal and immune activation. Adv Funct Mater. 2024;34(52):2410962.

[33] Cui M, Xu B, Wang L. Recent advances in multi-metallic-based nanozymes for enhanced catalytic cancer therapy. BMEMat. 2023;2(1):e12043.

[34] Barbero F, Russo L, Vitali M, Piella J, Salvo I, Borrajo ML, Busquets-Fité M, Grandori R, Bastús NG, Casals E, et al. Formation of the protein corona: The Interface between nanoparticles and the immune system. Semin Immunol. 2017;34:52–60. 29066063 10.1016/j.smim.2017.10.001

[35] Hu Q, Li J, Wang T, Xu X, Duan Y, Jin Y. Polyphenolic nanoparticle-modified probiotics for microenvironment remodeling and targeted therapy of inflammatory bowel disease. ACS Nano. 2024;18(20):12917–12932. 38720520 10.1021/acsnano.4c00830

[36] Jia M, Yi B, Chen X, Xu Y, Xu X, Wu Z, Ji J, Tang J, Yu D, Zheng Y, et al. Carbon dots induce pathological damage to the intestine via causing intestinal flora dysbiosis and intestinal inflammation. J Nanobiotechnol. 2023;21(1):1–17. 10.1186/s12951-023-01931-1 PMC10210306 37231475

[37] Solanki R, Rostamabadi H, Patel S, Jafari SM. Anticancer nano-delivery systems based on bovine serum albumin nanoparticles: A critical review. Int J Biol Macromol. 2021;193(Pt A):528–540. 34655592 10.1016/j.ijbiomac.2021.10.040

[38] Wang J, Zhang B. Bovine serum albumin as a versatile platform for cancer imaging and therapy. Curr Med Chem. 2018;25(25):2938–2953. 28292234 10.2174/0929867324666170314143335

[39] Sleep D. Albumin and its application in drug delivery. Expert Opin Drug Deliv. 2015;12(5):793–812. 25518870 10.1517/17425247.2015.993313

[40] Zhang S, Cho WJ, Jin AT, Kok LY, Shi Y, Heller DE, Lucy Lee Y-A, Zhou Y, Xie X, Korzenik JR, et al. Heparin-coated albumin nanoparticles for drug combination in targeting inflamed intestine. Adv Healthc Mater. 2020;9(16): e2000536. 32597571 10.1002/adhm.202000536 PMC7482138

[41] Jori C, Ahmad A, Kumar A, Kumar B, Ali A, Ali N, Tabassum H, Khan R. Bioactive chitosan-BSA Maillard-derived chrysin-loaded nanoparticles: A gastroprotective, biomucoadhesive approach for enhanced oral therapy in ulcerative colitis. Carbohydr Polym. 2025;359: 123537. 40306769 10.1016/j.carbpol.2025.123537

[42] Kim J, Zhang S, Zhu Y, Wang R, Wang J. Amelioration of colitis progression by ginseng-derived exosome-like nanoparticles through suppression of inflammatory cytokines. J Ginseng Res. 2023;47(5):627–637. 37720571 10.1016/j.jgr.2023.01.004 PMC10499592

[43] Stojanov S, Berlec A, Štrukelj B. The influence of probiotics on the Firmicutes/Bacteroidetes ratio in the treatment of obesity and inflammatory bowel disease. Microorganisms. 2020;8(11):1715. 33139627 10.3390/microorganisms8111715 PMC7692443

[44] Jiang K, Pang X, Li W, Xu X, Yang Y, Shang C, Gao X. Interbacterial warfare in the human gut: Insights from Bacteroidales’ perspective. Gut Microbes. 2025;17(1):2473522. 40038576 10.1080/19490976.2025.2473522 PMC11901371

[45] Zhang ZJ, Cole CG, Coyne MJ, Lin H, Dylla N, Smith RC, Pappas TE, Townson SA, Laliwala N, Waligurski E, et al. Comprehensive analyses of a large human gut Bacteroidales culture collection reveal species- and strain-level diversity and evolution. Cell Host Microbe. 2024;32(10):1853–1867. 39293438 10.1016/j.chom.2024.08.016 PMC11466702

[46] Price CE, Valls RA, Ramsey AR, Loeven NA, Jones JT, Barrack KE, Schwartzman JD, Royce DB, Cramer RA, Madan JC, et al. Intestinal Bacteroides modulates inflammation, systemic cytokines, and microbial ecology via propionate in a mouse model of cystic fibrosis. MBio. 2024;15(2): e0314423. 38179971 10.1128/mbio.03144-23 PMC10865972

[47] Hu H, Shao W, Liu Q, Liu N, Wang Q, Xu J, Zhang X, Weng Z, Lu Q, Jiao L, et al. Gut microbiota promotes cholesterol gallstone formation by modulating bile acid composition and biliary cholesterol secretion. Nat Commun. 2022;13(1):252. 35017486 10.1038/s41467-021-27758-8 PMC8752841

[48] Stummer N, Feichtinger RG, Weghuber D, Kofler B, Schneider AM. Role of hydrogen sulfide in inflammatory bowel disease. Antioxidants. 2023;12(8):1570. 37627565 10.3390/antiox12081570 PMC10452036

[49] Blachier F, Andriamihaja M, Larraufie P, Ahn E, Lan A, Kim E. Production of hydrogen sulfide by the intestinal microbiota and epithelial cells and consequences for the colonic and rectal mucosa. Am J Physiol Gastrointest Liver Physiol. 2021;320(2):G125–G135. 33084401 10.1152/ajpgi.00261.2020

