# Supplementary material for: Biomimetic Layered Double Hydroxide-Molybdenum Disulfide Encapsulated with Bovine Serum Albumin: A Multifaceted Nanotherapy for Inflammatory Bowel Disease
Source: Biomater Res. 2026 May 18;30:0369. doi: 10.34133/bmr.0369 (PMC13181168; doi:10.34133/bmr.0369)
Supplement: Supplementary 1 — Figs. S1 to S12 [file bmr.0369.f1.docx]

**Supporting information**

**Biomimetic LM@BSA Nanocomposite: A Multi-Faceted Nanotherapy for IBD**


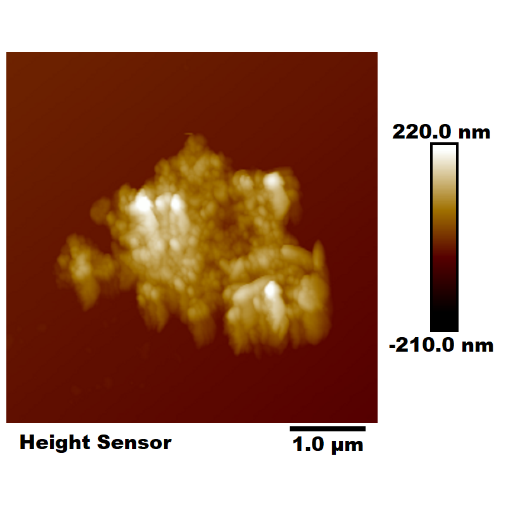


**Fig. S1.** AFM of LM@BSA nanocomposite.


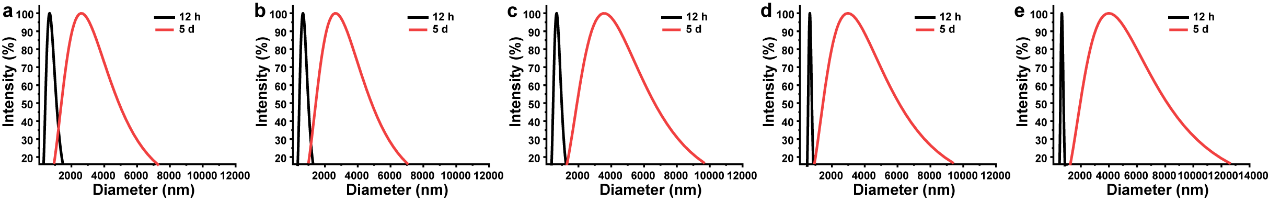


**Fig. S2.** DLS of LM nanosheets in (a) DMEM, (b) saline, (c) DI water, (d) SGF and (e) SIF, respectively.


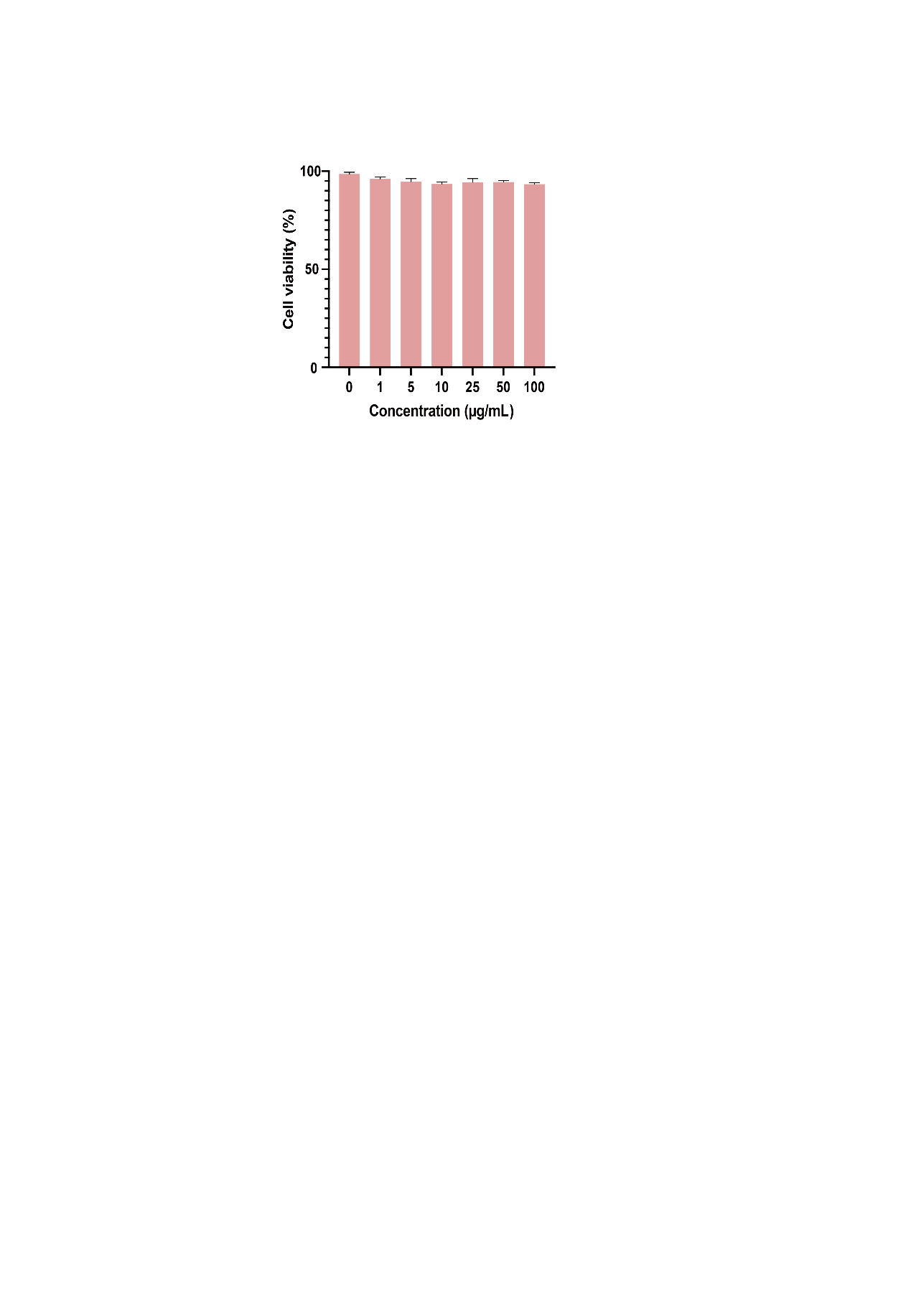


**Fig. S3.** The results of the CCK-8 assay indicated that LM@BSA nanocomposite exhibited minimal cytotoxicity.


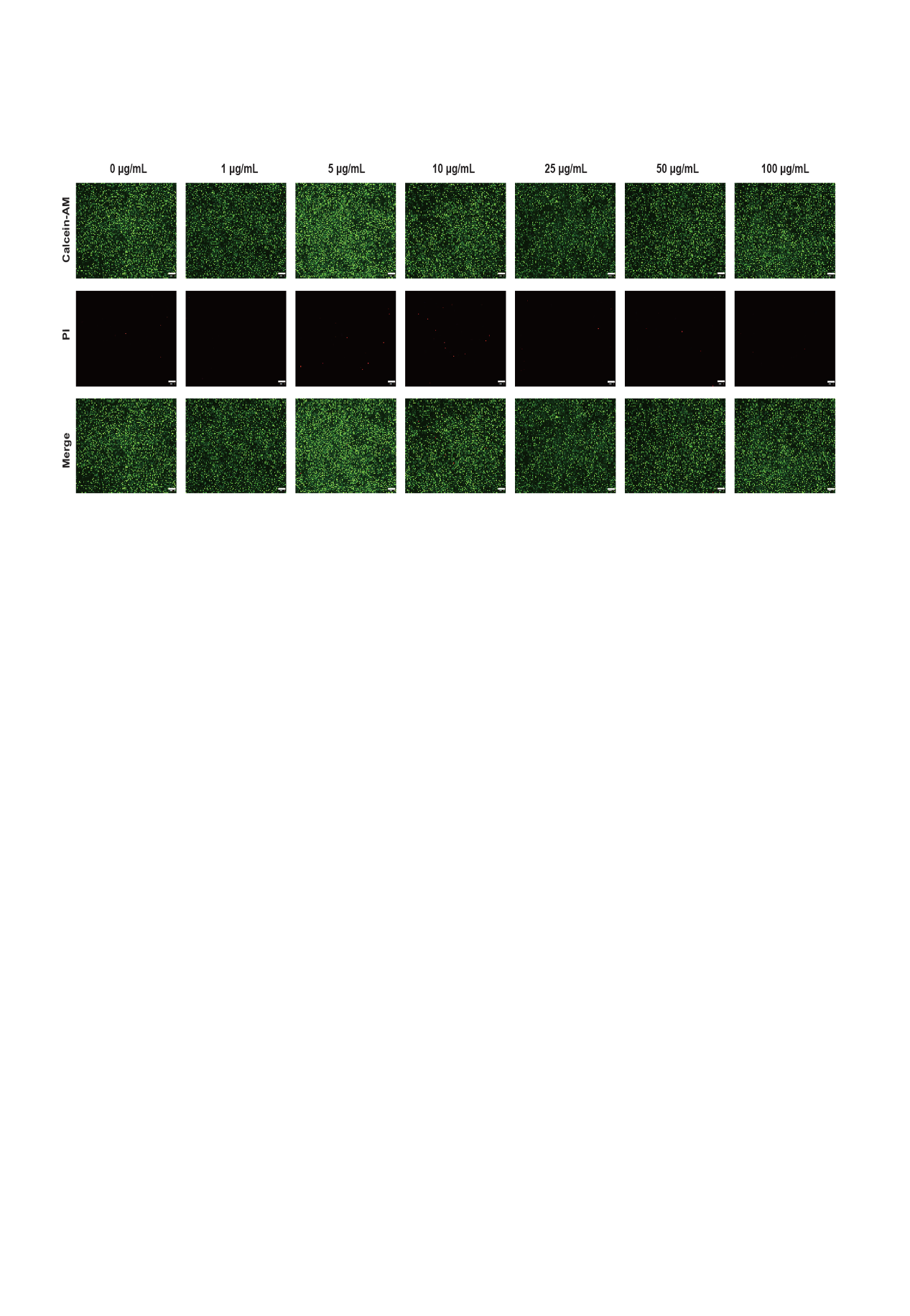


**Fig. S4.** The results of Live/Dead staining demonstrated the high biocompatibility of LM@BSA nanocomposite.


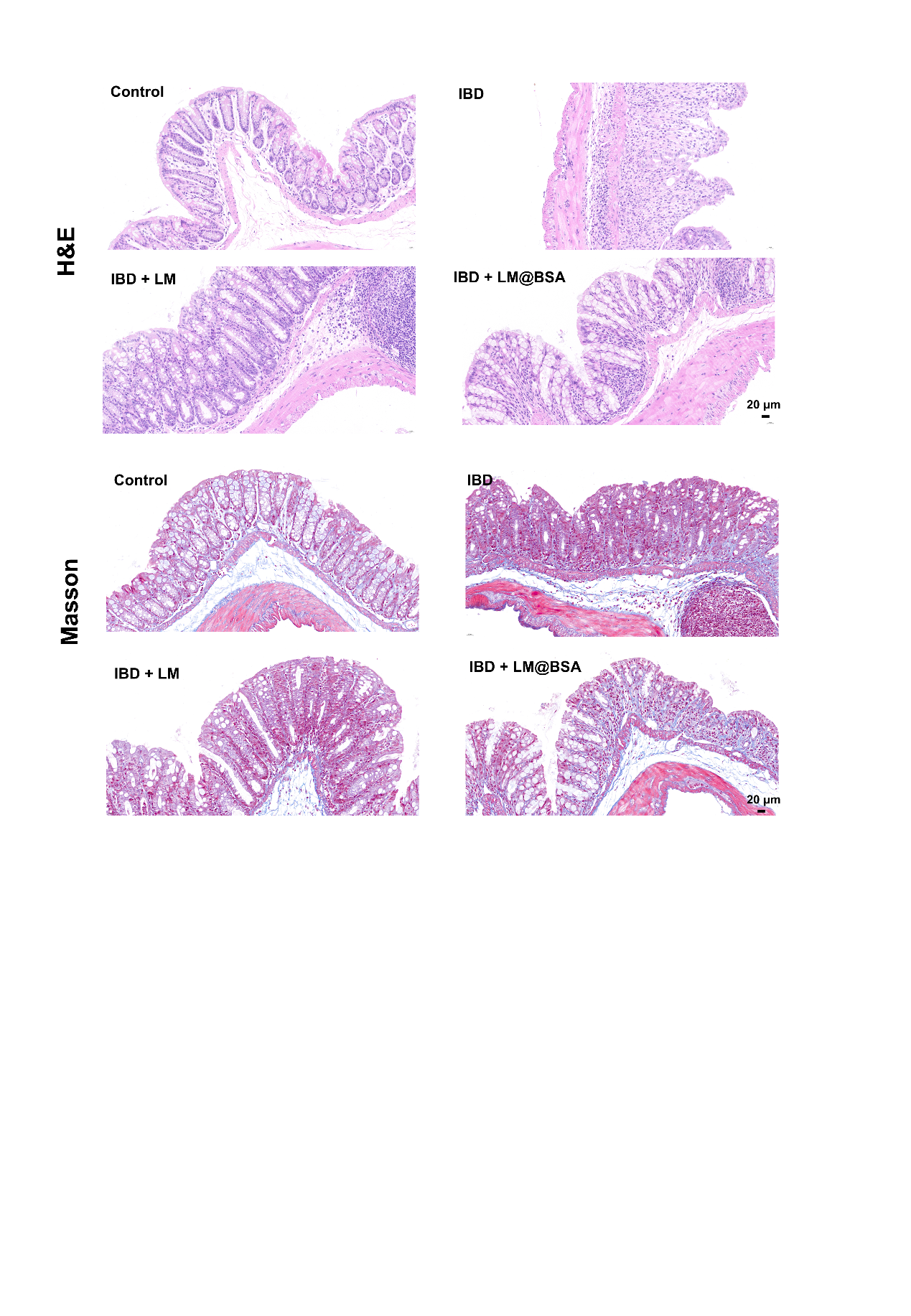


**Fig. S5.** Higher-magnification views of H&E staining (top) and Masson's trichrome staining (bottom).


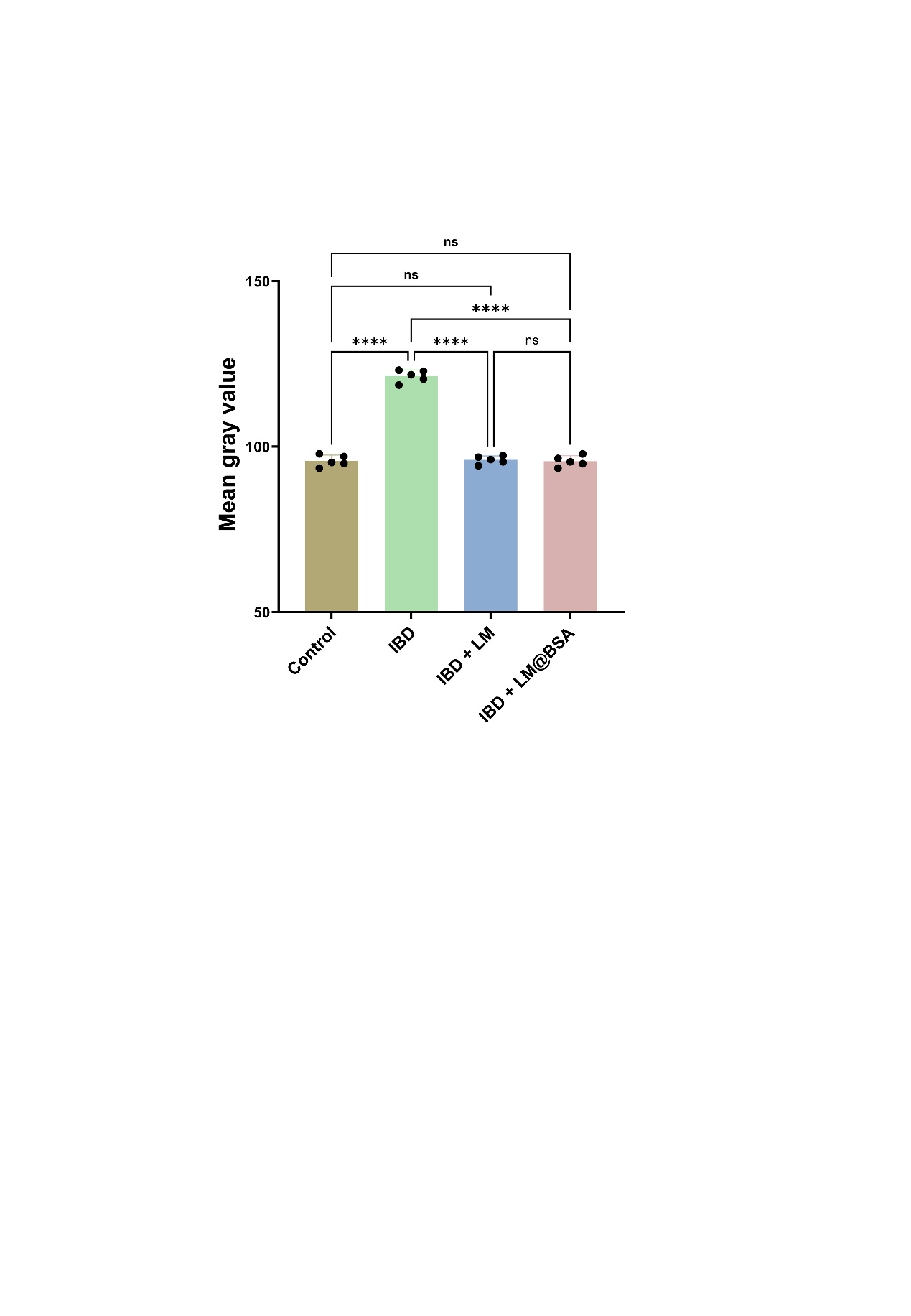


**Fig. S6.** Quantitative analysis of GPX4 immunohistochemical staining in colonic tissues. Statistical significance was determined by one-way ANOVA followed by Tukey’s post hoc test. Significance levels are denoted as **p* < 0.05, ***p* < 0.01, ****p* < 0.001, and *****p* < 0.0001; ns, not significant.


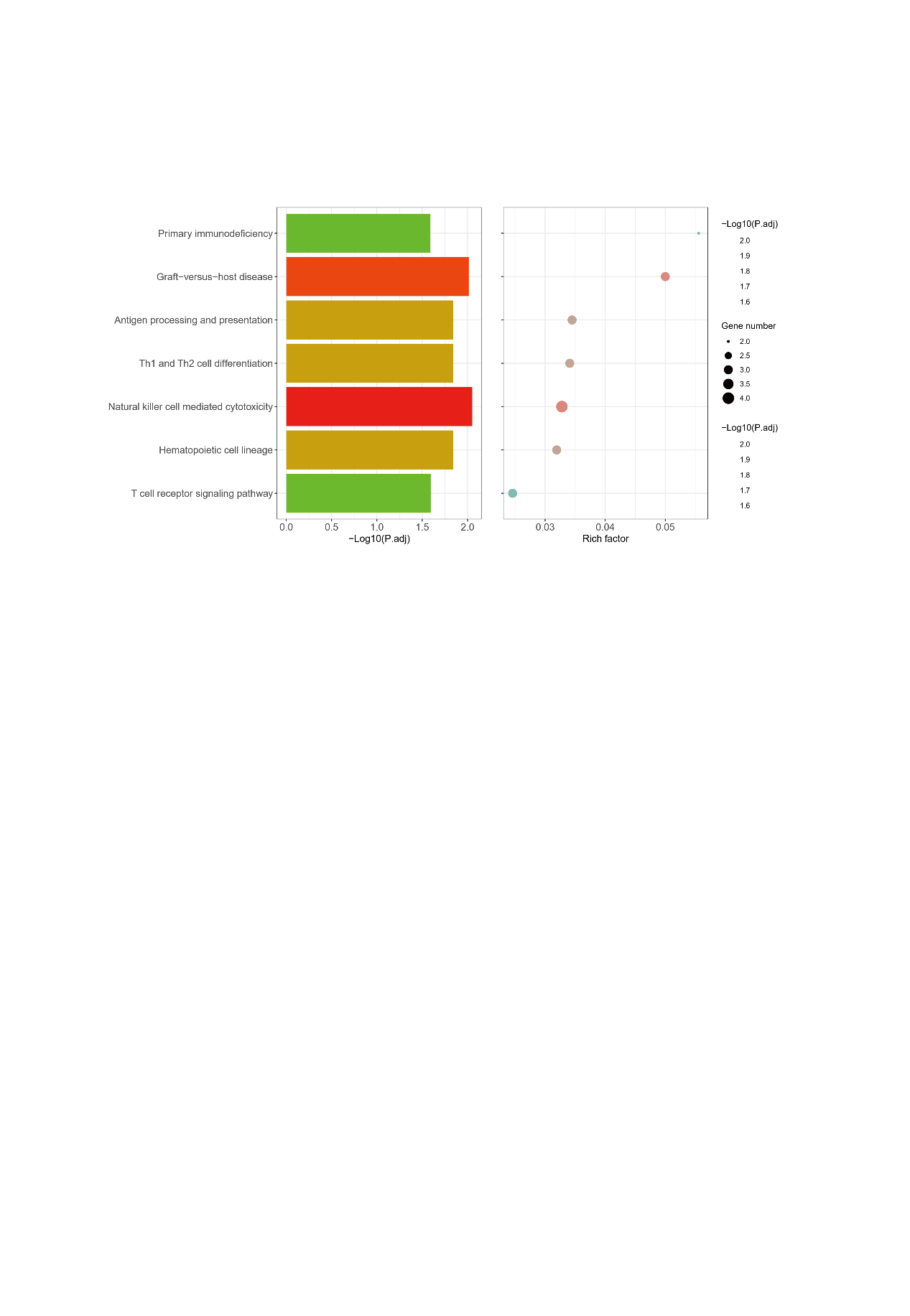


**Fig. S7.** The transcriptomic analysis revealed significant enrichment of immune-related pathways, including antigen processing and presentation, nature killer cell-mediated cytotoxicity, and Th1/Th2 cell differentiation.


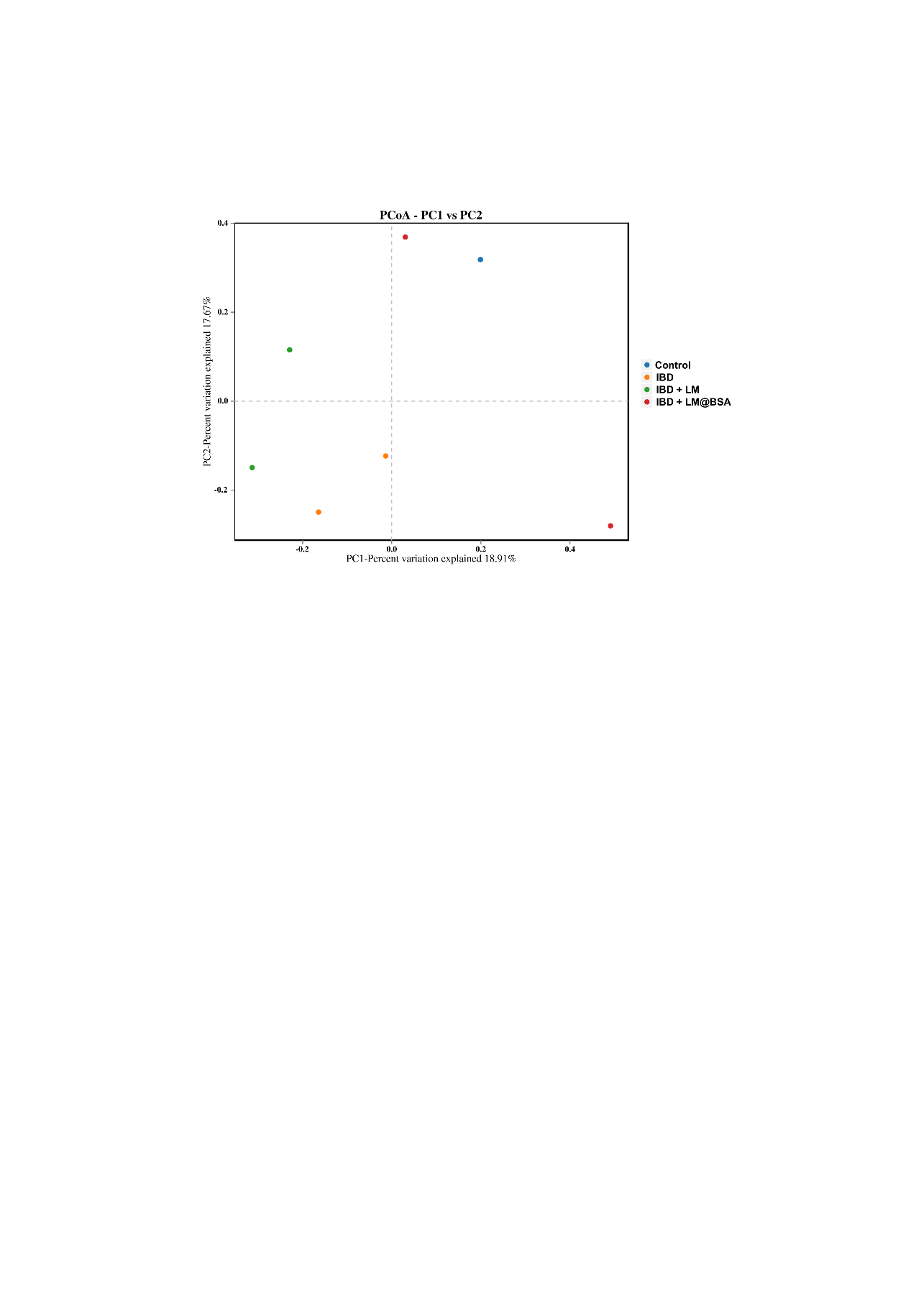


**Fig. S8.** PCoA revealed that DSS administration significantly disrupted the gut microbial community.


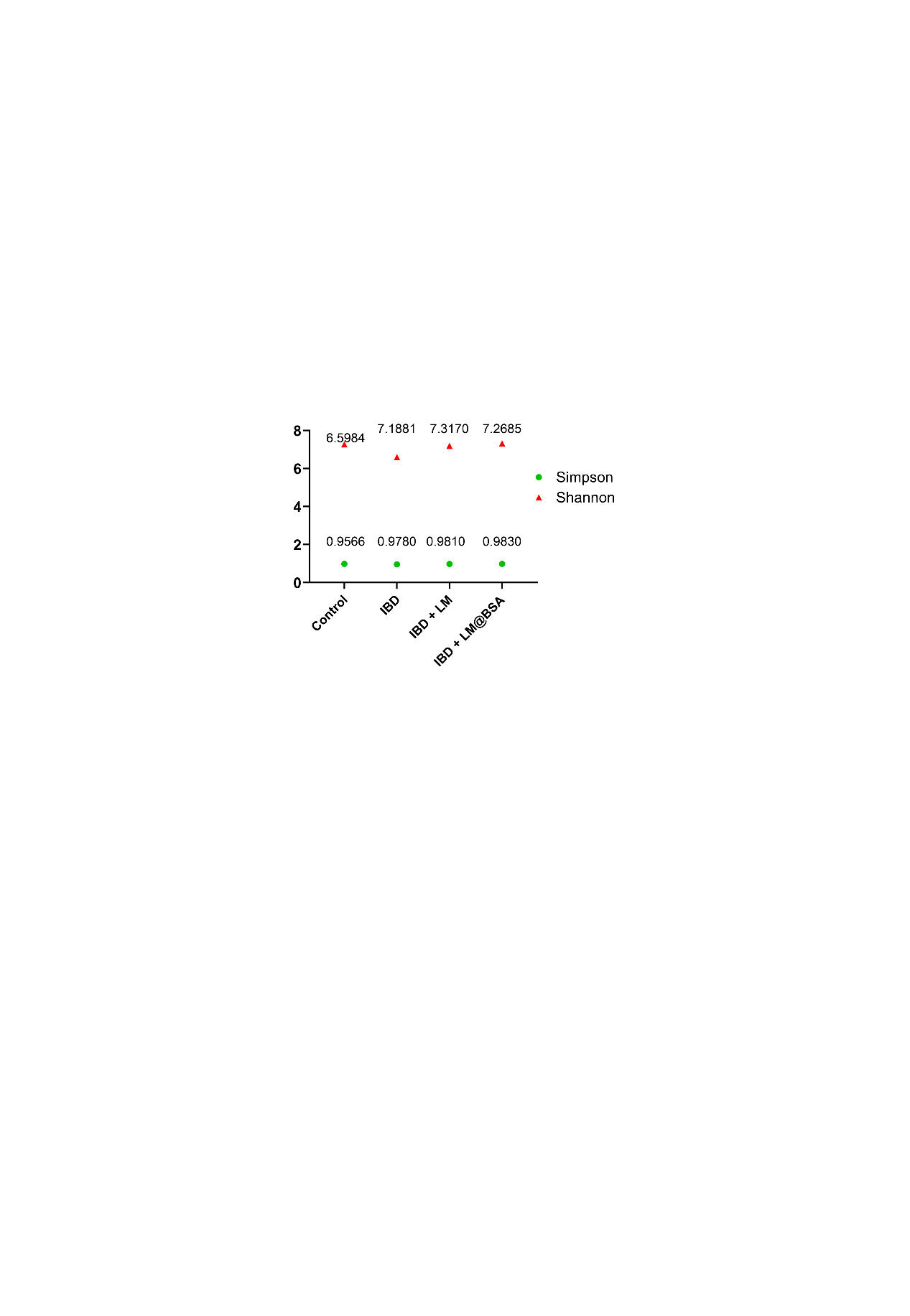


**Fig. S9.** The Simpson and Shannon indices of four groups showed that LM@BSA nanocomposite treatment restored microbial diversity to levels comparable to or exceeding those of the control group.


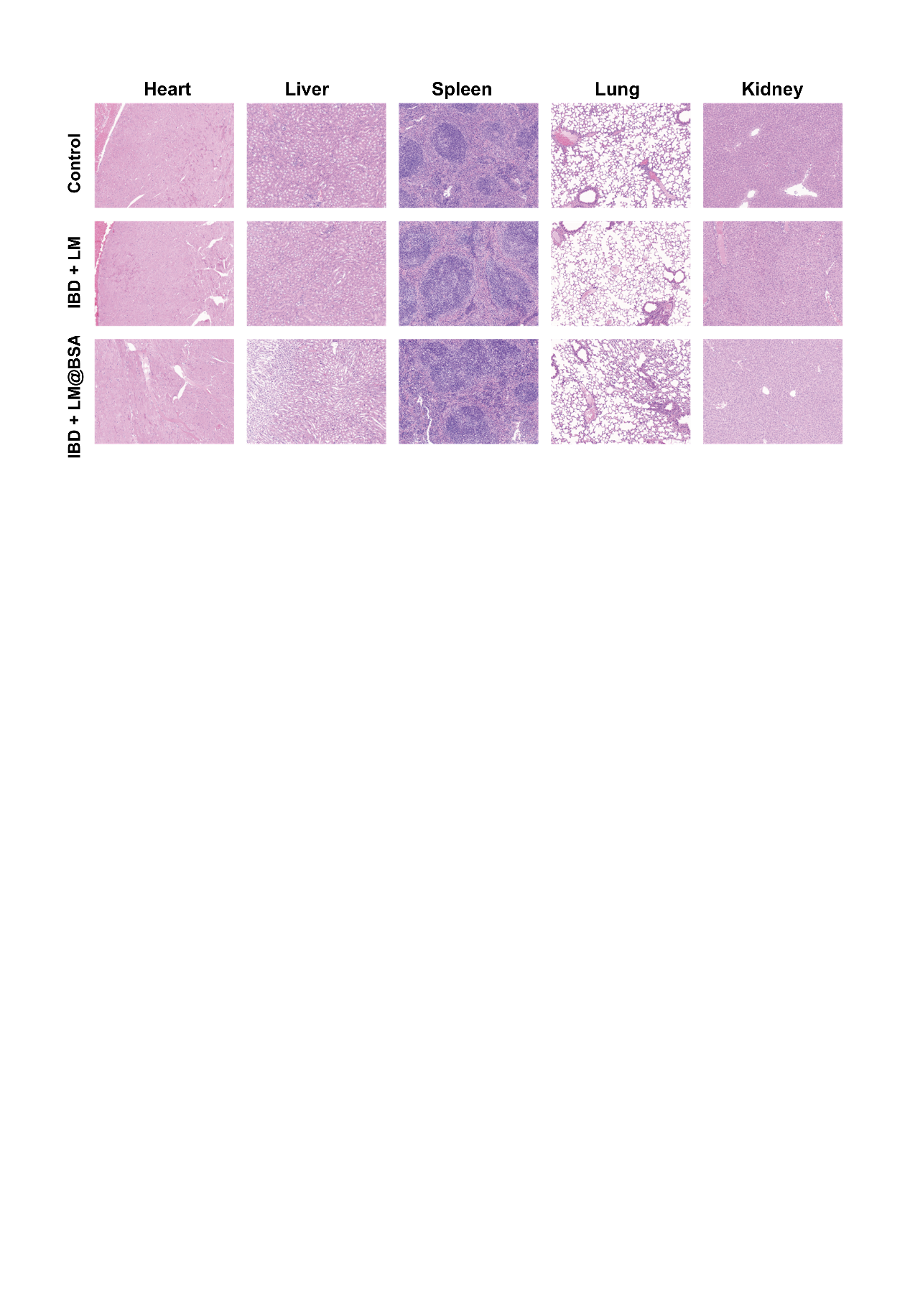


**Fig. S10.** H&E staining on heart, liver, spleen, lung, and kidney tissues showed that LM@BSA nanocomposite-treated mice had no obvious histological abnormalities.


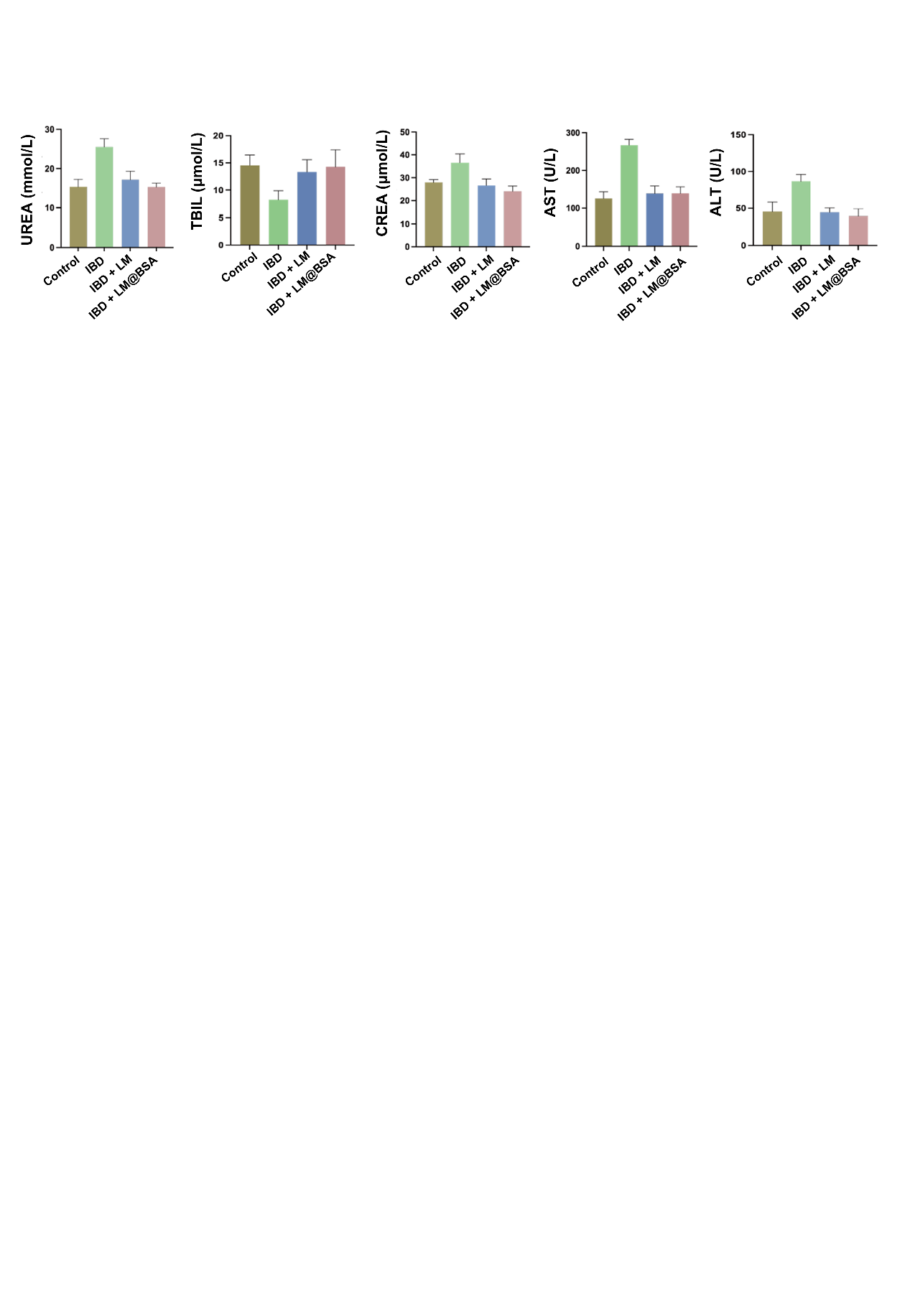


**Fig. S11.** Serum levels of UREA, TBIL, CREA, AST and ALT in the LM@BSA nanocomposite group confirmed that LM@BSA nanocomposite had excellent histocompatibility and biosafety.


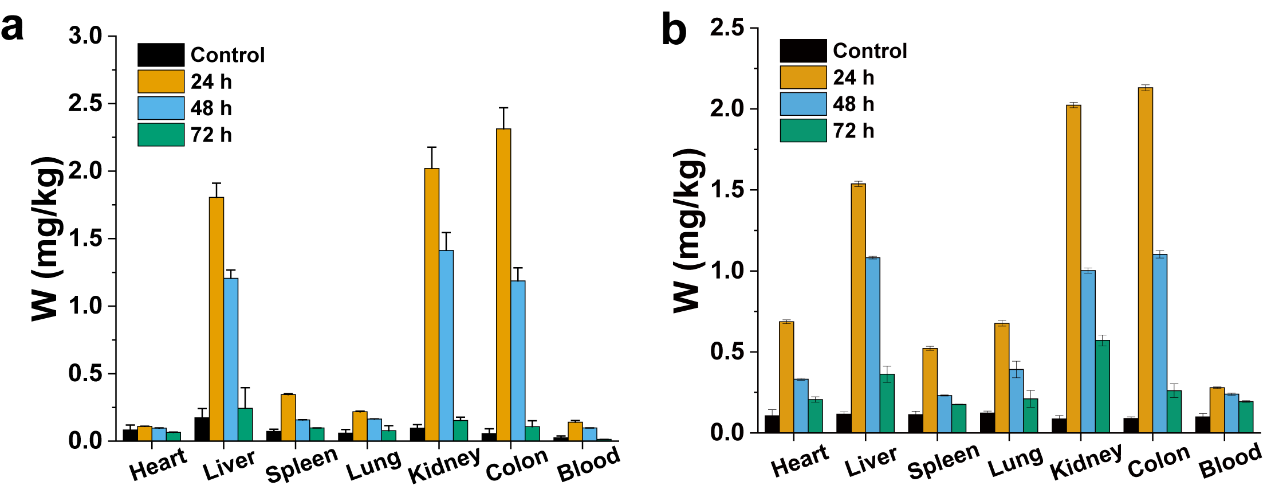


**Fig. S12.** (a) Mo and (b) Al biodistribution in mice after oral administration of LM@BSA nanocomposite.
